# Palmitoylation-dependent regulation of cardiomyocyte Rac1 signaling activity and minor effects on cardiac hypertrophy

**DOI:** 10.1016/j.jbc.2023.105426

**Published:** 2023-11-03

**Authors:** Tanya A. Baldwin, James P. Teuber, Yasuhide Kuwabara, Araskumar Subramani, Suh-Chin J. Lin, Onur Kanisicak, Ronald J. Vagnozzi, Weiqi Zhang, Matthew J. Brody, Jeffery D. Molkentin

**Affiliations:** 1 Department of Pediatrics, Cincinnati Children’s Hospital Medical Center, Cincinnati, Ohio, USA; 2 Department of Pharmacology, University of Michigan, Ann Arbor, Michigan, USA; 3 Department of Pathology, University of Cincinnati, Cincinnati, Ohio, USA; 4 Division of Cardiology, Department of Medicine, Consortium for Fibrosis Research & Translation, University of Colorado Anschutz Medical Campus, Aurora, Colorado, USA; 5 Laboratory of Molecular Psychiatry, Department of Mental Health, University of Münster, Münster, Germany; 6 Division of Cardiovascular Medicine, Department of Internal Medicine, University of Michigan, Ann Arbor, Michigan, USA

**Keywords:** cardiomyopathy, cardiac hypertrophy, cardiomyocyte, cell signaling, palmitoylation, S-acylation, proteomics, Ras homolog gene family, member A (RhoA), Ras-related C3 botulinum toxin substrate 1 (Rac1), G protein-coupled receptor, GTPase

## Abstract

S-palmitoylation is a reversible lipid modification catalyzed by 23 S-acyltransferases with a conserved zinc finger aspartate-histidine-histidine-cysteine (zDHHC) domain that facilitates targeting of proteins to specific intracellular membranes. Here we performed a gain-of-function screen in the mouse and identified the Golgi-localized enzymes zDHHC3 and zDHHC7 as regulators of cardiac hypertrophy. Cardiomyocyte-specific transgenic mice overexpressing zDHHC3 show cardiac disease, and S-acyl proteomics identified the small GTPase Rac1 as a novel substrate of zDHHC3. Notably, cardiomyopathy and congestive heart failure in zDHHC3 transgenic mice is preceded by enhanced Rac1 S-palmitoylation, membrane localization, activity, downstream hypertrophic signaling, and concomitant induction of all Rho family small GTPases whereas mice overexpressing an enzymatically dead zDHHC3 mutant show no discernible effect. However, loss of Rac1 or other identified zDHHC3 targets Gαq/11 or galectin-1 does not diminish zDHHC3-induced cardiomyopathy, suggesting multiple effectors and pathways promoting decompensation with sustained zDHHC3 activity. Genetic deletion of *Zdhhc3* in combination with *Zdhhc7* reduces cardiac hypertrophy during the early response to pressure overload stimulation but not over longer time periods. Indeed, cardiac hypertrophy in response to 2 weeks of angiotensin-II infusion is not diminished by *Zdhhc3/7* deletion, again suggesting other S-acyltransferases or signaling mechanisms compensate to promote hypertrophic signaling. Taken together, these data indicate that the activity of zDHHC3 and zDHHC7 at the cardiomyocyte Golgi promote Rac1 signaling and maladaptive cardiac remodeling, but redundant signaling effectors compensate to maintain cardiac hypertrophy with sustained pathological stimulation in the absence of zDHHC3/7.

Cardiac hypertrophy is an adaptive growth response of the heart whereby cardiomyocytes enlarge to maintain cardiac output. Although initially beneficial, cardiac hypertrophy often becomes pathological, resulting in adverse remodeling and decompensation that ultimately further impinge on cardiac function and accelerate the progression to heart failure (1, 2, 3). Thus, there is great interest in the delineation of intracellular signaling mechanisms that facilitate pathological cardiac growth that could potentially be inhibited to delay or prevent the transition from cardiac hypertrophy to heart failure.

Diverse intracellular signaling pathways participate in cardiac pathologic hypertrophy and heart failure (1, 2, 3, 4). Pathological signaling in cardiomyocytes is often transduced from the sarcolemma (plasma membrane) by GTPases that activate downstream intracellular signaling cascades (5, 6, 7, 8, 9, 10). Activation of small GTPases is dynamically regulated by guanine nucleotide dissociation inhibitors (GDIs), GTPase activating proteins, and guanine nucleotide exchange factors (11, 12, 13, 14). Additionally, some small GTPases such as H-Ras, N-Ras, and Ras-related C3 botulinum toxin substrate 1 (Rac1) undergo S-palmitoylation or S-acylation, a reversible lipid modification on cysteine residues that governs their dynamic association with the plasma membrane and subsequent activation of downstream effectors (15, 16, 17). Moreover, certain GTPase regulatory proteins, including p63 RhoGEF (18) and the regulator of G-protein signaling proteins that function as GTPase activating proteins for heterotrimeric Gα subunits (19, 20), are S-palmitoylated, providing another layer of S-palmitoylation–dependent control of signaling by G proteins. However, the enzymes controlling fatty acylation of GTPases and the consequences of S-palmitoylation on signaling by small GTPases are not well-established, particularly in the context of cardiomyocyte signaling in hypertrophy and heart failure.

Cardiomyocyte-specific overexpression of RhoA or Rac1 causes cardiac failure in mice (10, 21), and RhoGTPase signaling is activated in murine cardiomyopathy (7, 22) and human heart failure (23). Conversely, deletion of RhoA is detrimental in response to chronic pressure overload (24) whereas deletion of Rac1 is beneficial (25). Rac1 is also an essential mediator of reactive oxygen species generation in the heart through regulation of the NADPH oxidase-2 (Nox2) complex (26, 27, 28) and is required for cardiac hypertrophy and oxidative stress in response to angiotensin II (6). Importantly, impairment of Rac1 activity and oxidative stress are primary mechanisms of statin-mediated cardioprotection in animal models (6, 29, 30), and statin treatment ameliorates Rac1 activation, NADPH oxidase activity, and reactive oxygen species production in the failing human heart (23). However, the mechanisms that modulate Rac1 signaling in the heart remain ill-defined.

The dynamic nature of protein S-palmitoylation provides a regulatory mechanism akin to protein phosphorylation, with diverse effects on protein localization and function. Small GTPases and G protein α subunits undergo rapid cycles of S-palmitoylation and depalmitoylation to elicit sustained signaling activity (31, 32), implicating S-palmitoylation as a critical control point for intracellular signal transduction. There are 23 zinc finger Asp-His-His-Cys (zDHHC) S-acyltransferases (encoded by the *Zdhhc* genes) in mammals that catalyze S-palmitoylation (33, 34, 35), which is reversed predominantly by the cytosolic depalmitoylases, acyl protein thioesterase 1 and 2 (APT-1 and APT-2) (36), and α/β-hydrolase domain containing 17 family proteins (ABHD17A/B/C) (37). zDHHC S-acyltransferases are polytopic transmembrane proteins, many of which localize to the endoplasmic reticulum or Golgi apparatus with some also residing at the plasma membrane, endomembrane system, or intracellular vesicles (33, 38). Despite some common substrates among different zDHHC enzymes, there is generally strong selectivity and substrate specificity imparted by recruitment domains on the cytoplasmic tails of zDHHCs (36, 39, 40). Even among Golgi-localized zDHHCs, there is specified recruitment of substrates by their cognate zDHHC enzyme (41, 42). Thus, S-palmitoylation is a tightly controlled regulatory mechanism that underlies intracellular signal transduction *via* dynamic targeting of proteins to membrane microdomains. However, studies of zDHHC enzymes and S-palmitoylation in the heart are largely limited to investigation of ion channel regulation and electrophysiology (40, 43), and roles of S-palmitoylation in cardiomyocyte signal transduction and hypertrophy and heart failure remain understudied.

Here, we performed an *in vivo* screen using recombinant adeno-associated virus (AAV)-mediated overexpression, which identified the closely related Golgi-localized enzymes zDHHC3 and zDHHC7 as inducers of cardiac maladaptation, decompensation, and heart failure. While zDHHC3 is expressed in the heart (38, 40), much of the prior work in the field focused on its functions in neurons (44, 45, 46, 47). We found that Rac1 is a novel substrate of zDHHC3 using an unbiased proteomic approach and that cardiomyocyte-specific transgenic mice overexpressing *Zdhhc3*, but not an enzymatically dead mutant, develop lethal dilated cardiomyopathy. Indeed, zDHHC3 transgenic mice develop cardiomyopathy and heart failure with enhanced Rac1 S-palmitoylation and plasma membrane localization, along with activation of other Rho GTPase family members. Genetic deletion of *Zdhhc3* alone or in combination with *Zdhhc7* does not impair baseline cardiac structure-function and pathological hypertrophy in response to chronic pressure overload or angiotensin-II stimulation but does alter the initiation of hypertrophy in response to acute pressure overload stimulation. These studies identify zDHHC3 and zDHHC7 S-acyltransferase activity at the cardiomyocyte Golgi as a regulator of RhoGTPase activity that is sufficient to promote cardiac maladaptation and heart failure but not overtly required for cardiac remodeling in response to pressure overload or angiotensin-II.

## Results

### Overexpression of zDHHC3 or zDHHC7 induces dilated cardiomyopathy

**Figure 1:**
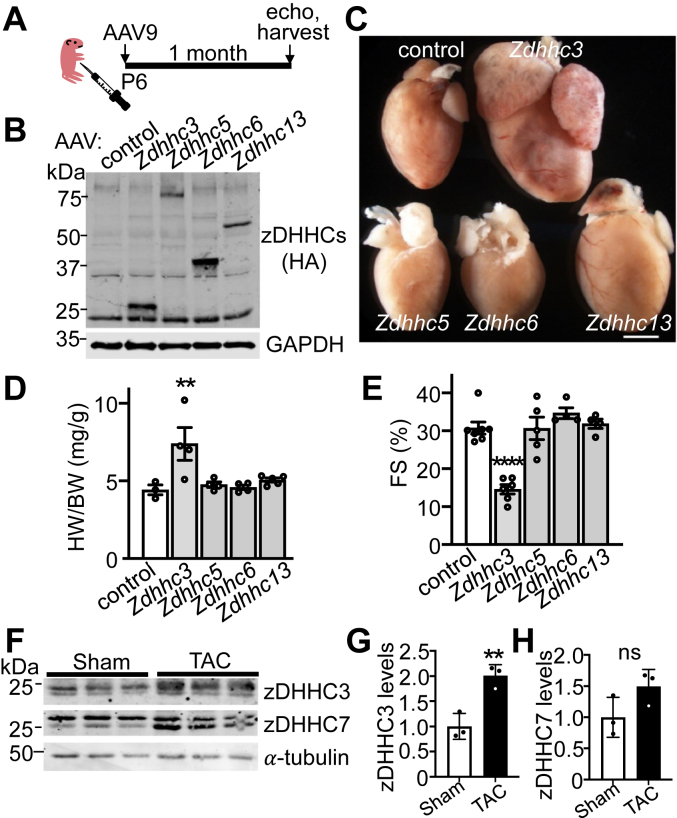
**Enhanced activity of the Golgi-resident enzyme zDHHC3 causes cardiomyopathy.***A*, experimental design schematic and (*B*) Western blotting for AAV9-mediated overexpression of HA-tagged zDHHC enzymes in the heart analyzed at 30 days of age from prior recombinant AAV9-Zdhhc injection in 6-day-old pups (P6). GAPDH is used as tissue processing and Western loading control. *C*, gross morphology of hearts from mice shown in *B* (Scale bar represents 1 mm). *D*, heart weight-to-body weight ratios (HW/BW) of the indicated groups of mice, n = 3 to 5. One way ANOVA (*p* = 0.005) with pairwise comparison test of control compared to Zdhhc3 (*p* < 0.0056), Zdhhc5 (*p* = 0.88), Zdhhc6 (*p* = 0.88), and Zdhhc13 (*p* = 0.78). *E*, fractional shortening (FS) as assessed by echocardiography in mice with cardiac overexpression of the indicated zDHHC enzymes at 30 days of age. n = 4 to 7. One way ANOVA (*p* < 0.0001) with pairwise comparison of control compared to Zdhhc3 (*p* = 0.0001), Zdhhc5 (*p* = 0.97), Zdhhc6 (*p* = 0.40), and Zdhhc13 (*p* = 0.91). *F–H*, Western blotting (*F*) and quantification (*G* and *H*) of zDHHC3 and zDHHC7 protein levels in mouse hearts after 8 weeks of pressure overload stimulation (TAC; transverse aortic constriction) compared to sham controls. α-tubulin is a tissue processing and western loading control. Error bars throughout the figure panels represent mean ± SEM. ∗∗*p* < 0.01, ∗∗∗∗*p* < 0.0001. AAV, adeno-associated virus.

We performed an *in vivo* screen by overexpressing several zDHHC enzymes in the heart with adeno-associated virus serotype 9 (AAV9). Pups were injected with AAV9 at postnatal day 6 to induce cardiac expression of the Golgi-localized enzymes zDHHC3 and zDHHC13 as well as the plasma membrane enzyme zDHHC5 and the endoplasmic reticulum–localized enzyme zDHHC6, protein expression, cardiac morphology and function were assessed 1 month later (Fig. 1, *A*–*C*). Enhanced expression of the Golgi-resident S-acyltransferase zDHHC3 resulted in a profound cardiomyopathy that was not observed with the overexpression of these other zDHHC enzymes tested (Fig. 1, *C–E*). Cardiac overexpression of *Zdhhc3* complementary DNA (cDNA) resulted in cardiac enlargement including ventricular and atrial dilation (Fig. 1*C*), cardiac hypertrophy (Fig. 1*D*), and substantial cardiac dysfunction indicative of cardiomyopathy (Fig. 1*E*). The most homologous S-acyltransferase to zDHHC3 is another Golgi-localized enzyme, zDHHC7, and AAV9-mediated overexpression of this enzyme in the heart similarly induced cardiomyopathy within 3 weeks (Fig. S1, *A–E*). These data collectively suggest that the activity of the Golgi-localized zDHHC3 and zDHHC7 enzymes promote pathogenic intracellular signaling that results in cardiac hypertrophy and decompensation. Importantly, endogenous protein levels of zDHHC3 are increased in the adult mouse heart in response to pressure overload–induced hypertrophic stimulation (Fig. 1, *F–H*), suggesting a physiologic role of S-palmitoylation mediated by zDHHC3 and/or zDHHC7 in cardiac maladaptation. Notably, although zDHHC3 and zDHHC7 are appreciably expressed in many cardiac cell types, including myocytes and fibroblasts (data not shown) (48, 49), RNA-seq of isolated cardiomyocytes revealed upregulation of *ZDHHC3* and *ZDHHC7* transcripts in human dilated cardiomyopathy (50), implicating a pathophysiologic role for cardiomyocyte zDHHC3/7 in the heart.

**Figure 2:**
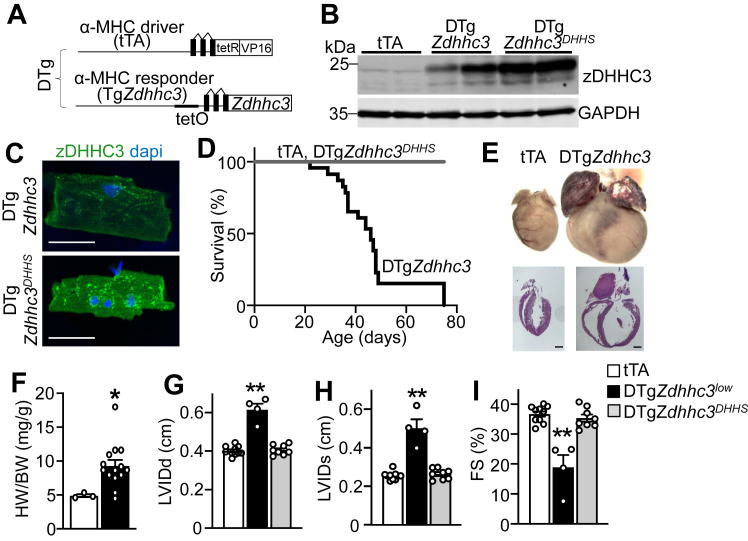
**Cardiac-specific overexpression of zDHHC3 results in cardiomyopathy and premature lethality.***A*, schematic diagram of the bi-transgenic “tet-off” inducible system used to overexpress *Zdhhc3* in the heart. *B* and *C*, cardiomyocyte-specific transgenic expression of zDHHC3 or zDHHC3^DHHS^ protein 2-months after doxycycline chow removal to induce transgene expression in the indicated high-expressing lines of mice. *B*, Western blot for zDHHC3 expression in cardiac lysates. GAPDH is run as a loading and protein processing control. *C*, immunocytochemistry on adult isolated cardiomyocytes demonstrating Golgi localization pattern for both zDHHC3 and enzymatically-dead zDHHC3^DHHS^ protein (*green*) from double transgenic (DTg) hearts after 2 months of transgene expression (3 months of age). Scale bar represents 50 μm. *D*, Kaplan-Meier survival curve showing mortality in DTg mice with perinatal cardiomyocyte-specific expression of zDHHC3 *versus* the enzymatically dead control DTg mice. n = 14 tTA, 21 DTg*Zdhhc3*, and 13 DTg*Zdhhc3*^*DHHS*^. Survival curve log-rank test (*p* < 0.0001). *E*, whole hearts imaged together for comparison with no scale bar shown and histology (H&E-stained) of transgenic hearts at 7 weeks of age, scale bar = 500 μm. *F*, HW/BW at 4 weeks of age in the indicated groups of mice. n = 3 to 14, unpaired *t* test compared to tTA (*p* = 0.037). *G–I*, echocardiographic measurement of (*G*), diastolic left ventricular inner diameter in diastole (LVIDd), one way ANOVA (*p* < 0.0001) with pairwise comparison of control (tTA) compared to DTg*Zdhhc3* (*p* < 0.0001) and DTg*Zdhhc3*^*DHHS*^ (*p* = 0.75). *H*, systolic LVID (LVIDs), one way ANOVA (*p* < 0.0001) with pairwise comparison of control (tTA) compared to DTg*Zdhhc3* (*p* < 0.0001) and DTg*Zdhhc3*^*DHHS*^ (*p* = 0.65). *I*, FS% at 6 to 8 weeks of age. One way ANOVA (*p* < 0.0001) with pairwise comparison of control (tTA) compared to DTg*Zdhhc3* (*p* < 0.0001) and DTg*Zdhhc3*^*DHHS*^ (*p* = 0.52). n = 4 to 10. Error bars throughout the figure panels represent mean ± SEM. ∗*p* < 0.05, ∗∗*p* < 0.01. DTg, double transgenic; HW/BW, heart weight-to-body weight; tTA, tetracycline transactivator.

To further evaluate functions of zDHHC3-mediated S-palmitoylation *in vivo*, we generated mice with cardiomyocyte-specific overexpression of *Zdhhc3* using a binary and inducible system consisting of the tetracycline transactivator (tTA) protein and the tet operator downstream of a modified α-myosin heavy chain (αMHC) promoter (51) such that double transgenic mice (DTg*Zdhhc3*) containing both the tTA and *Zdhhc3* transgenes express zDHHC3 protein in the heart if doxycycline (Dox) is not present in the diet (“tet-off” system) (52) (Fig. 2, *A* and *B*). As an additional control, we generated cardiac-specific transgenic mice that overexpress an enzymatically dead zDHHC3 protein containing a Cys-to-Ser point mutation in its enzymatic DHHC domain (DTg*Zdhhc3*^*DHHS*^) (Fig. 2, *A* and *B*). Western blotting of heart extracts from adult mice showed abundant overexpression of each protein in the heart compared with tTA controls (Fig. 2*B* and S2, *A* and *B*). Immunocytochemistry in isolated adult cardiomyocytes revealed the expected Golgi localization pattern for overexpressed wildtype and transferase-dead zDHHC3 proteins (Fig. 2*C*). Transgenic mice on a normal diet with overexpression of *Zdhhc3* starting around birth (when ventricular αMHC expression begins), but not mice overexpressing the enzymatically dead *Zdhhc3*^*DHHS*^ mutant, exhibited substantial mortality in young adulthood due to severe dilated cardiomyopathy with a median survival of 6 weeks of age in low expressing lines (Fig. 2, *D* and *E*) and around 3 weeks of age in a high-expressing line (data not shown). Gross morphological and histological analyses revealed dramatic cardiac enlargement and ventricular and atrial dilation in DTg*Zdhhc3* hearts (Fig. 2*E*) and heart weight-to-body weight (HW/BW) ratios confirmed significant cardiac hypertrophy (Fig. 2*F*). Cardiac function and structure were evaluated by echocardiography, which revealed significant left ventricular dilation (Fig. 2*G*), systolic dysfunction (Fig. 2*H*), and impaired cardiac contraction and cardiomyopathy (Fig. 2*I*) in mice with cardiomyocyte-specific overexpression of *Zdhhc3* but not in mice expressing the *Zdhhc3*^*DHHS*^ mutant (Fig. 2, *G–I*). These results demonstrate that enhanced zDHHC3 S-acyltransferase activity in cardiomyocytes causes severe lethal dilated cardiomyopathy.

To examine the role of zDHHC3 in the adult heart, we kept transgenic mice on a Dox-containing diet until weaning to keep expression off and then switched them to normal lab chow to induce transgene expression (Fig. S3*A*). Overexpression of *Zdhhc3* for the first time in the adult heart did not result in immediate cardiomyopathy as observed with perinatal overexpression of *Zdhhc3* in cardiomyocytes (Fig. 2). However, *Zdhhc3* overexpression in adult cardiomyocytes resulted in lethality within 7 or 10 months of transgene expression in the high- and low-expressing lines, respectively (Fig. S3*B*). Mortality in DTg*Zdhhc3* mice was preceded by clinical symptoms of congestive heart failure, including dyspnea and peripheral edema (Fig. S3*C*) as well as cardiac hypertrophy and ventricular and atrial dilation (Fig. S3, *C–F*). Cardiac functional assessment by echocardiography revealed systolic impairment (Fig. S3*F*) and cardiac dysfunction (Fig. S3*G*) in DTg*Zdhhc3* mice prior to mortality. Lower-expressing lines exhibited an identical phenotype with a delayed onset and progression of disease (Fig. S3, *C–G*). In contrast, overexpression of the *Zdhhc3*^*DHHS*^ transferase–dead mutant in the adult heart did not cause cardiac hypertrophy, adverse remodeling, or cardiomyopathy at any age examined (Fig. S3, *D–G*). Taken together, these data demonstrate the expression of zDHHC3 S-acyltransferase activity in adult cardiomyocytes causes congestive heart failure.

### Rac1 is a novel substrate of zDHHC3

To identify zDHHC3 substrates that could underlie cardiac maladaptation, we employed a quantitative and site-specific proteomic approach to sequence peptides containing S-palmitoylated cysteine residues. We generated stable NIH3T3 cell lines that overexpress *Zdhhc3* or enhanced green fluorescent protein (eGFP) as a control and performed stable isotope labeling with amino acids in cell culture (SILAC) for quantitative mass spectrometry sequencing (53). S-palmitoylated proteins were purified from 3T3-*Zdhhc3* and 3T3-eGFP cells by Acyl resin-assisted capture (Acyl-RAC) (54), trypsin-digested on thiopropyl sepharose, and eluted to release bound peptides containing the S-palmitoylation sites for mass spectrometry sequencing. 3T3-*Zdhhc3* cells were labeled with “heavy” lysine and arginine while 3T3-eGFP controls were labeled with media containing normal isotopic lysine and arginine (“light”) such that peptides identified with increased heavy:light ratios (H:L) exhibit increased S-palmitoylation in *Zdhhc3*-overexpressing cells. Altogether, we identified 82 unique proteins and 101 unique peptides containing H:L ratios above 1.2, which we also categorized separately for known cardiac signaling effectors (Table S1 and Supporting Data Files 1 and 2), suggesting regulation by zDHHC3 activity. Peptides sequenced included the previously reported zDHHC3 modification sites on phosphatidylinositol 4-kinase IIa (PI4K2α) (55, 56) as well as previously reported S-palmitoylation sites on caveolin-2 (57), Rac1 (17), scribble (58), and Trappc3 (59). An identical strategy was also performed to compare mouse embryonic fibroblast cultures that were wildtype *versus* cultures deleted for *Zdhhc3*, which identified additional putative targets of zDHHC3 including Gα_q/11_, an established substrate of zDHHC3 and zDHHC7 (60), as the signaling protein with the most prominent reduction in S-palmitoylation in *Zdhhc3*-deleted cells (Table S1).

**Figure 3:**
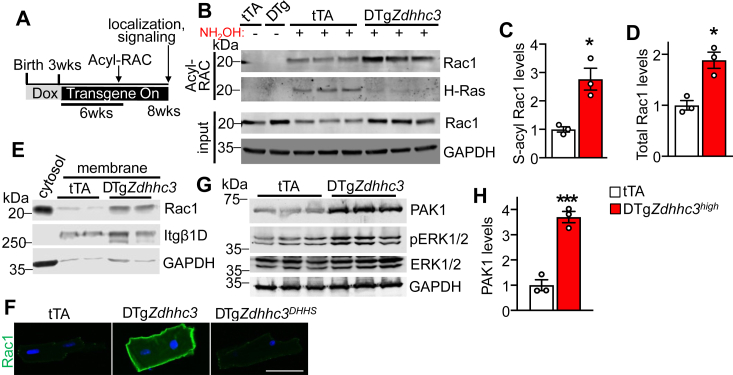
**Rac1 S-palmitoylation, membrane localization, and effector signaling are regulated by zDHHC3 in the heart.***A*, experimental schematic for assessment of the impacts of zDHHC3 overexpression on protein S-palmitoylation and signaling *in vivo*. Mice were bred on doxycycline chow to suppress transgene expression until being weaned onto normal lab chow to induce transgene expression in adulthood. Hearts were then harvested after 6 weeks of transgene expression in the adult heart for analyses of protein S-palmitoylation in panels (*B–D*) or after 8 weeks of transgene expression for evaluation of Rac1 membrane localization and signaling in panels (*E–H*). *B*, immunoblotting for the indicated S-palmitoylated proteins purified by Acyl-RAC. (−) indicates negative controls lacking NH_2_OH treatment. Input for the assay is also shown. *C* and *D*, quantification of (*C*) S-palmitoylated, unpaired *t* test (*p* = 0.01) and (*D*) total Rac1 protein levels normalized to GAPDH from (*B*), unpaired *t* test (*p* = 0.009). n = 3. *E*, immunoblotting for the indicated membrane proteins isolated from transgenic hearts. GAPDH is a protein loading and tissue processing control. *F*, immunocytochemistry for endogenous Rac1 (*green*) in cardiomyocytes isolated from transgenic hearts expressing zDHHC3 or the enzymatically dead zDHHC3^DHHS^ mutant protein. Scale bar represents 50 μm. *G*, immunoblotting for the Rac1 effector PAK1 and phosphorylation of its substrate, ERK1/2, in transgenic hearts and (*H*), quantification of PAK1 protein levels, unpaired *t* test (*p* = 0.001). n = 3. Error bars throughout the figure panels represent mean ± SEM. ∗*p* < 0.05, ∗∗∗*p* = 0.001. ERK, extracellular signal–regulated kinase; Itgβ1D, integrin β1D; PAK, p21-activated kinase.

The proteomic screen suggests zDHHC3 directly modifies Rac1 at Cys-178 (Table S1), which is critical for its activation and localization to specific plasma membrane microdomains involved in actin cytoskeletal reorganization (17). Cys-178 of Rac1 is located in its C-terminal membrane-targeting domain that also contains the classical prenylated-CAAX motif required for processing and trafficking of all small GTPases (61). Importantly, S-palmitoylation–dependent regulation of Rac1 has not been evaluated in cardiomyocytes or *in vivo* to date. To determine if zDHHC3 S-palmitoylates Rac1 in the heart, we performed Acyl-RAC assays to purify S-palmitoylated proteins from transgenic hearts followed by immunoblotting, where we observed a substantial increase in S-palmitoylated Rac1 in zDHHC3-overexpressing hearts (Figs. 3, *A–C* and S4, *A–D*) concomitant with upregulation of total Rac1 protein levels (Figs. 3, *B* and *D* and S4*C*). H-Ras S-palmitoylation was reduced in *Zdhhc3*-overexpressing hearts (Fig. 3*B*), indicating specificity of zDHHC3 for modification of Rac1 in cardiomyocytes. Notably, induction of Rac1 S-palmitoylation in DTg*Zdhhc3* hearts occurred within 2 weeks of transgene expression, prior to the development of cardiac hypertrophy and heart failure (Fig. S4), suggesting that modification of Rac1 may be a proximal mechanism underlying zDHHC3 activity–induced cardiac pathology. Immunoblotting of membrane fractions from transgenic hearts after 8 weeks of transgene expression demonstrated a substantial increase in membrane-associated Rac1 with *Zdhhc3* overexpression (Fig. 3*E*). Finally, immunostaining of isolated myocytes from transgenic hearts similarly revealed a dramatic enhancement in plasma membrane–associated Rac1 relative to tTA and DTg*Zdhhc3*^*DHHS*^ controls (Fig. 3*F*).

Western blotting analyses of signaling molecules downstream of Rac1 revealed a substantial increase in the expression of the Rac1 effector, p21-activated kinase 1 (PAK1) (62, 63), in *Zdhhc3*-overexpressing hearts (Fig. 3, *G* and *H*) as well as increased phosphorylation of extracellular signal–regulated kinases 1 and 2 (ERK1/2) (Fig. 3*G*), which are activated by PAK1 (64, 65, 66) and function as transducers of cardiac hypertrophy (67, 68, 69). Thus, zDHHC3-mediated S-palmitoylation enhances Rac1 translocation to the sarcolemma and downstream activation of PAK1 and ERK1/2.

### Overexpression of Zdhhc3 enhances RhoGTPase signaling

**Figure 4:**
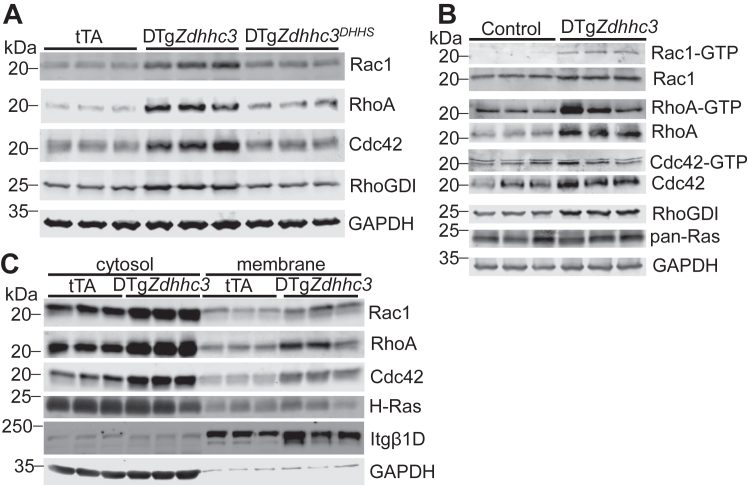
**Enhanced zDHHC3 activity induces signaling by all Rho family small GTPases in the heart.** Western blotting for (*A*) the indicated RhoGTPase family members in hearts overexpressing zDHHC3 or the enzymatically dead zDHHC3^DHHS^ mutant protein (*high line*, 2 months of transgene expression in the adult heart). tTA control hearts were also used. *B*, active (-GTP) and total levels of Rho small GTPases family members in transgenic hearts overexpressing *Zdhhc3* (low line, five months of transgene expression in adult heart). Controls are nontransgenic or single transgenic tTA or *Zdhhc3* littermates of DTg*Zdhhc3* mice overexpressing zDHHC3 protein. *C*, cytosolic and membrane protein fractions isolated from tTA control and transgenic hearts and blotted for the indicated proteins. GAPDH is used throughout as a tissue processing and protein loading control. Itgβ1D, integrin β1D (to show membrane fraction purity). tTA, tetracycline transactivator.

Remarkably, *Zdhhc3* overexpression in the transgenic mouse heart, but not the enzymatically dead mutant, had a profound effect on all RhoGTPase family proteins, eliciting an increase in the abundance of not just Rac1 but also RhoA, Cdc42, and RhoGDI (Fig. 4*A*). There was also a concomitant elevation in the levels of active, GTP-bound RhoA in addition to Rac1 (Fig. 4*B*). These data are consistent with enhanced Rac1 membrane translocation (Fig. 3, *E* and *F*) and effector signaling (Fig. 3, *G* and *H*) observed in *Zdhhc3*-overexpressing hearts. RasGTPase expression was unaffected by zDHHC3 activity (Fig. 4, *B* and *C*), suggesting the specificity for RhoGTPase signaling. Indeed, protein levels of Rac1, RhoA, and Cdc42, but not H-Ras, were elevated in both cytoplasmic and membrane fractions isolated from *Zdhhc3*-overexpressing hearts compared to tTA controls (Fig. 4*C*). RhoGDI serves as a master regulator of RhoGTPase signaling homeostasis by regulating the abundance, activity, and localization of all RhoGTPase family proteins (11, 12, 70). We observed that protein levels of RhoGDI were also substantially increased in *Zdhhc3*-overexpressing hearts (Fig. 4, *A* and *B*), suggesting a broad-spectrum effect of zDHHC3 activity on RhoGTPase signaling.

### Cardiac pathology by Zdhhc3 is not rescued by gene deletion of selected S-palmitoylated targets

To directly examine the hypothesis that *Zdhhc3* overexpression in the heart drives hypertrophy and cardiomyopathy through Rac1 signaling induction, we utilized *Rac1*-loxP-targeted (*Rac1*^*f/f*^) mice crossed with αMHC promoter–driven Cre transgenic animals to establish a cardiomyocyte-specific deletion of *Rac1* in the heart with zDHHC3 overexpression (DTg*Zdhhc3 Rac1*^*f/f-αMHCcre*^). However, deletion of *Rac1* in the heart did not alter the progression of *Zdhhc3* overexpression–driven cardiomyopathy (Fig. S5, *A* and *B*). Our proteomic screen also identified Gα protein subunits q and 11 (Gα_q_ and Gα_11_) as targets of zDHHC3 palmitoylation (Table S1), which support the previous findings of zDHHC3 and zDHHC7 knockdown resulting in reduced Gα_q_ and Gα_11_ palmitoylation and membrane localization (60). Moreover, activation of small RhoGTPases including RhoA and Rac1 lie downstream of G protein–coupled receptors that mediate Gα_q_ and Gα_11_ activation (71). We utilized mice lacking *Gna11* with *Gnaq*-loxP–targeted mice that were crossed with the *Nkx2.5cre* allele to generate double *Gnaq/Gna11* (72)–deleted mice that were crossed with mice containing the *Zdhhc3* transgene (DTg*Zdhhc3* Gα_q_^f/f-Nkx2.5cre^ G_11_^−/−^). However, no rescue of the *Zdhhc3* overexpression–driven cardiomyopathy was observed in mice lacking Gα_q_ and Gα_11_ in the heart (Fig. S5, *C* and *D*).

Our proteomic screen also identified multiple palmitoylation sites on the small GTPase regulatory protein galectin-1 with *Zdhhc3* overexpression (Table S1). The structure of galectin-1 is analogous to the prenyl-binding pocket of RhoGDI that interacts with RhoGTPases (73) and galectin-1 is known to function as a GDI-like chaperone to regulate H-Ras activity and membrane localization (73, 74, 75), suggesting that zDHHC3-mediated S-palmitoylation of galectin-1 may serve as a molecular switch to control RhoGTPase signaling. To determine if zDHHC3 palmitoylates galectin-1 in the heart, we performed Acyl-RAC on transgenic hearts and immunoblotted for galectin-1. We observed an increase in palmitoylated galectin-1 as well as total galectin-1 protein with cardiomyocyte overexpression of *Zdhhc3* (Fig. S6*A*). Biochemical fractionation (Fig. S6*B*) and immunocytochemistry (Fig. S6*C*) demonstrated robust localization of galectin-1 to the cardiomyocyte membrane in *Zdhhc3*-overexpressing hearts but not hearts overexpressing the enzymatically dead *Zdhhc3* mutant. However, *Lgals1* (galectin-1) gene–deleted mice containing the *Zdhhc3* transgene (DTg*Zdhhc3 Lgals1*^*−/−*^) did not show alterations in the enhanced expression of RhoGTPases (RhoA, Rac1, Cdc42, or RhoGDI) (Fig. S6*D*). More importantly, deletion of *Lgals1* did not impact cardiomyopathy and the reduction in fractional shortening caused by *Zdhhc3* overexpression (Fig. S6*E*).

### Deletion of Zdhhc3/7 impairs initiation of cardiac hypertrophy in response to pressure overload and Rac1 S-palmitoylation levels

**Figure 5:**
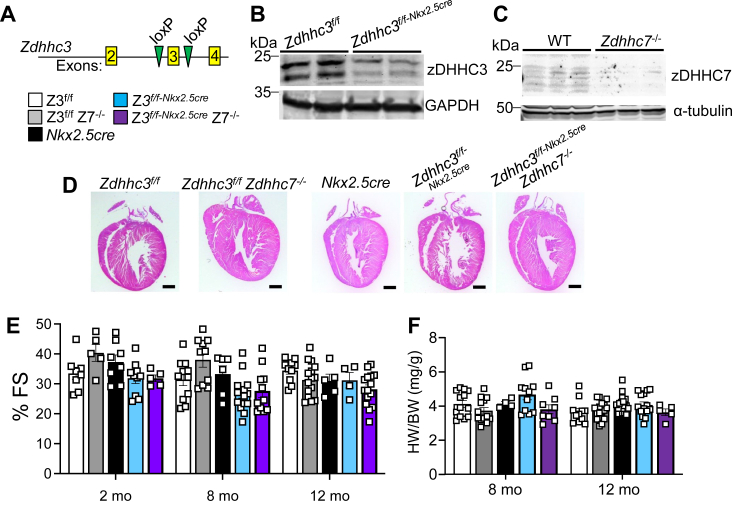
**Deletion of *Zdhhc7* or cardiac-specific deletion of *Zdhhc3* does not alter heart characteristics.***A*, schematic of *Zdhhc3* gene deletion and placement of loxP sites for tissue-selective deletion. *B*, Western blotting for zDHHC3 from hearts of the indicated 2-month-old mice, with GAPDH serving as a loading control. *C*, Western blot of zDHHC7 from heart protein extract of the indicated mice at 2 months of age, with α-tubulin serving as a loading control from cardiac lysates. *D*, H&E-stained cardiac histological sections at 8 months of age in the indicated mice. Scale bar represents 500 μm. *E*, echocardiography measured FS% assessed at 2-, 8-, and 12-months in the indicated mice. Two-way ANOVA showed no interaction (*p* = 0.11). *F*, HW/BW in the indicated mice at the specified time points. Two-way ANOVA showed no interaction (*p* = 0.42). Error bars throughout the figure panels represent mean ± SEM. Z3, *Zdhhc3* targeted. Z7, *Zdhhc7* targeted. HW/BW, heart weight-to-body weight.

To further probe the physiological role of zDHHC3 in the heart, we generated *Zdhhc3*-loxP(f)–targeted mice (Zdhhc3^f/f^) crossed with the *Nkx2.5cre* allele to establish cardiomyocyte-specific deletion of the *Zdhhc3* gene (*Zdhhc3*^*f/f-Nkx2.5cre*^) (Fig. 5, *A* and *B*). We also examined *Zdhhc7* gene–deleted (*Zdhhc7*^*−/−*^) mice (Fig. 5*C*) that were crossed with the cardiac-specific *Zdhhc3*-deleted mice to generate double nulls (*Zdhhc3*^*f/f-Nkx2.5cre*^ *Zdhhc7*^*−/−*^). We first assessed changes in baseline function and morphology with deletion of *Zdhhc7* by comparing *Zdhhc3*^*f/f*^, *Zdhhc3*^*f/f*^ *Zdhhc7*^*−/−*^, *Nkx2.5cre, Zdhhc3*^*f/f-Nkx2.5cre*^, and *Zdhhc3*^*f/f-Nkx2.5cre*^ *Zdhhc7*^*−/−*^ mice (Fig. 5*D*). No changes in cardiac function from 2 to 12 months were observed as measured by echocardiography, HW/BW ratio analysis, or morphology between any of the groups (Fig. 5, *E* and *F*). Taken together, zDHHC3 and zDHHC7 are not overtly required for baseline structure-function of the mouse heart separately or in combination.

**Figure 6:**
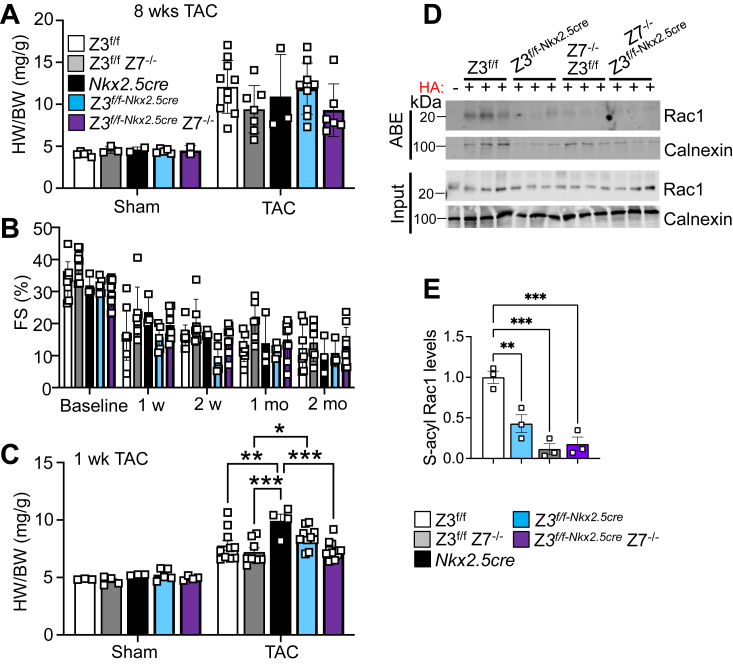
**Deletion of *Zdhhc3* and *Zdhhc7* significant attenuates the initiation of the hypertrophic response but not chronic hypertrophy in response to 8 weeks of pressure overload.***A*, two-month-old mice of the indicated groups were subjected to transverse aortic constriction (TAC) or a sham surgical procedure for 8 weeks (2 months) and HW/BW was assessed. Two-way ANOVA showed a main effect of TAC (*p* < 0.0001) but no interaction between the genotypes (*p* = 0.61). *B*, echocardiography measured FS% at baseline and at 1 week, 2 weeks, 1 month, and 2 months post TAC *versus* sham in the indicated groups of mice. Two-way ANOVA showed a main effect of TAC *versus* Sham (*p* < 0.001) but no interaction was observed between the genotypes (*p* = 0.68). *C*, two-month-old mice were subjected to TAC for 1 week and HW/BW was assessed compared to sham controls in the indicated groups of mice. Sham n = 3 to 5, TAC n = 4 to 12. Two-way ANOVA showed a main effect of TAC (*p* < 0.0001) and genotype (*p* = 0.005) but no interaction (*p* = 0.12). *D* and *E*, Western blotting (*D*) and quantification (*E*) of the indicated S-palmitoylated proteins purified by Acyl-biotin exchange (ABE) relative to input (shown below) from cardiac lysates generated from the indicated groups of mice at 8 months of age. (−) indicates negative controls lacking hydroxyl amine (HA) treatment. n = 3 to 7. Calnexin was used as a control. Error bars throughout the figure panels represent mean ± SEM. ∗*p* < 0.05, ∗∗*p* < 0.01, ∗∗∗*p* < 0.001. Z3, *Zdhhc3* targeted. Z7, *Zdhhc7* targeted. HW/BW, heart weight-to-body weight.

We observed increased endogenous protein levels of zDHHC3 in the heart in response to 8 weeks of pressure overload–induced hypertrophic stimulation (Fig. 1*F*) and a prior study revealed upregulation of *Zdhhc3* transcript levels after 1 week of pressure overload (50). To determine whether deletion of *Zdhhc3* and/or *Zdhhc7* contribute to the development of hypertrophy and pathological signaling, we subjected single- and double-targeted mice to transverse aortic constriction (TAC). Both single- (*Zdhhc3*^*f/f-Nkx2.5cre*^ or *Zdhhc7*^*−/−*^) and double-deleted (*Zdhhc3*^*f/f-Nkx2.5cre*^ *Zdhhc7*^*−/−*^) mice showed similar increases in HW/BW ratios after 8 weeks of TAC compared with control groups (Fig. 6*A*) and a similar reduction in cardiac function over these time points (Fig. 6*B*). Moreover, cardiac hypertrophy in response to 2 weeks of chronic angiotensin-II infusion was not significantly altered by deletion of *Zdhhc3* or *Zdhhc7* alone or in combination (Fig. S7). We also surveyed mRNA levels of other *Zdhhc* genes and found no alterations in single- or double-deleted mice (Fig. S8). However, we did observe a phenotype with pressure overload in which *Zdhhc3*^*f/f-Nkx2.5cre*^ *Zdhhc7*^*−/−*^ mice showed a modest but significant reduction in the degree of cardiac hypertrophy with 1 week of TAC, as assessed by HW/BW measurement (Fig. 6*C*). Pairwise comparison showed no changes in sham mice between genotypes, while *Zdhhc3*^*f/f-Nkx2.5cre*^*, Zdhhc7*^*−/−*^ and double null mice showed a reduction in HW/BW over 1 week of TAC compared with *Nkx2.5cre* controls. (Fig. 6*C*). Consistent with these results, the palmitoylation levels of Rac1 are reduced in the hearts of both single- (*Zdhhc3*^*f/f-Nkx2.5cre*^ and *Zdhhc7*^*−/−*^) and double (*Zdhhc3*^*f/f-Nkx2.5cre*^ *Zdhhc7*^*−/−*^)-targeted mice at 8 months of age (Fig. 6, *D* and *E*). Taken together, these results suggest that zDHHC3/7 activity facilitates the cardiac hypertrophic response during the first week of TAC and these enzymes can dramatically alter Rac1 activity, but there after, other compensatory effectors compensate to drive heart growth.

## Discussion

S-palmitoylation plays critical roles in the pathophysiology of cancer (76, 77, 78, 79, 80, 81), inflammation (82, 83, 84, 85), peripheral artery disease (86), and thrombosis (87), yet few roles have been established for this posttranslational modification in the pathogenesis of cardiac hypertrophy and heart failure. Despite S-palmitoylation of essential cardiac signal transducing proteins (*i.e.*, ⍺- and β-adrenergic receptors (88, 89)), endothelin receptors (90, 91, 92), Gαq (60, 93), and Gαs (93, 94), little is known of the functional effects of these lipid modifications and the enzymes that mediate them. Here, we surveyed zDHHC S-acyltransferase enzymes and observed that activity of zDHHC3 and zDHHC7 at the cytoplasmic surface of the Golgi promotes hypertrophic signaling and cardiomyopathy *in vivo*. Further investigation identified Rac1 as a target of zDHHC3 in the heart, and both *Zdhhc3* and *Zdhhc7* were shown to be important in the initiation of cardiac hypertrophy with pressure overload stimulation. To date, only zDHHC9 has been implicated in cardiac pathophysiology and adaptation, where it represses atrial natriuretic peptide release *via* S-palmitoylation of Rab3gap1 and impairment of the Rab3 GTPase cycle, and sustained zDHHC9 overexpression in cardiomyocytes ultimately results in dilated cardiomyopathy in late adulthood (95).

Rac1 plays fundamental roles in cardiac homeostasis and pathophysiology and is necessary and sufficient to induce cardiac hypertrophy (6, 21, 96) and arrhythmia (62, 97). Overexpression of constitutively active Rac1 results in lethal dilated cardiomyopathy (21) and arrhythmogenesis (98), while loss of Rac1 in cardiomyocytes ameliorates angiotensin II–induced cardiac hypertrophy and oxidative stress (6). Rac1 canonically signals from lamellipodia and membrane ruffles in nonmuscle cells (63, 99, 100) but how Rac1 functions in cardiomyocytes with a relatively static cytoskeleton, limited migration and proliferation, and unique sarcolemmal signaling domains is not well understood. Here, we uncovered a novel regulatory mechanism governing spatiotemporal control of cardiomyocyte Rac1 signaling activity through zDHHC3-mediated S-palmitoylation.

Functions of small GTPases are regulated by their translocation to cellular membranes where they modulate signaling by effector molecules that ultimately impact a host of cellular processes including cell growth, proliferation, and migration (13, 101). All Ras superfamily small GTPases (which include Rho and Rab family GTPases) undergo prenylation on their C terminus, the irreversible modification of cysteines with an unsaturated isoprenyl fatty acid, which is critical for their processing, trafficking, and ultimately membrane association (102, 103, 104). Rho family GTPases (RhoA/C, Rac1, Cdc42) are primarily regulated by RhoGDI, a Rho-specific chaperone molecule that binds the prenylated C terminus of RhoGTPases to regulate their delivery to and extraction from sites of action at cell membranes (11, 12, 70). We uncovered zDHHC3-regulated S-palmitoylation of Rac1 at Cys-178, adjacent to its C-terminal polybasic region and prenylated CAAX motif, which has been shown to target Rac1 to higher ordered cholesterol-rich membrane microdomains with increased activation (17). These data suggest that zDHHC3-mediated Rac1 S-palmitoylation compartmentalizes Rac1 at distinct sarcolemmal signaling microdomains that likely underlie pathological remodeling and hypertrophy. zDHHC3-overexpressing hearts exhibit induction of the Rac1 effector kinase, PAK1, and phosphorylation of ERK1/2, regulators of hypertrophic cardiac growth (64, 66, 67, 68). These data collectively suggest a working hypothesis whereby zDHHC3 activity at the cardiomyocyte Golgi S-palmitoylates Rac1 to promote its sarcolemmal translocation and signaling activity along with induction of all small GTPases of the Rho family and RhoGDI, which is associated with congestive heart failure in zDHHC3 transgenic mice that also phenocopies cardiac-specific overexpression of RhoA or Rac1 (10, 21). However, genetic deletion of Rac1 in the heart is unable to rescue cardiac maladaptation observed with zDHHC3 overexpression, and *Zdhhc3* heart-specific null mice still show robust cardiac hypertrophy over 8 weeks of TAC stimulation or 2 weeks of Ang-II infusion. These data suggest that zDHHC3-regulated signaling is more critically involved in the initiation of the hypertrophic response, but that other pathways compensate over longer periods of stimulation. Moreover, *Zdhhc3* overexpression in the heart undoubtedly induces the cardiac hypertrophic response through many downstream effectors as suggested in Table S1; hence it seems unlikely that deletion of a single key effector gene (*i.e.*, Rac1) would be sufficient to attenuate the greater response over longer periods of time.

Other critical effectors have also been shown to mediate maladaptive signaling downstream of zDHHC3/7 in diverse cellular systems. For example, disruption of signal transducer and activator of transcription 3 (Stat3) S-palmitoylation cycling by genetic deletion of *Zdhhc7* or pharmacological inhibition of APT-2 ameliorates inflammatory gene expression and colitis in an animal model of inflammatory bowel disease (82). Thus, palmitoylation cycling of soluble signaling proteins can provide a regulatory mechanism to garner sustained signaling and activation of downstream transduction circuitry, and the Golgi-localized zDHHC proteins appear to be critical in such signaling. Here, we observed that zDHHC3 activity in cardiomyocytes regulates S-palmitoylation of Rac1 and consequently its translocation to the sarcolemma, GTP-loading, and activation of downstream effectors, and zDHHC3 is indispensable for maintaining Rac1 S-palmitoylation in the aged heart. These data suggest that zDHHC3-mediated regulation of Rac1 S-palmitoylation cycling induces pathogenic signaling, which could represent a new therapeutic vantage point in cardiomyopathy and heart failure.

In summary, our data are the first to demonstrate a critical role for dynamic S-palmitoylation in the heart as a regulator of pathologic signal transduction that leads to hypertrophy and maladaptive ventricular remodeling. Interestingly, statin drugs commonly prescribed to treat cardiovascular disease repress membrane localization, activation, and abundance of Rac1 in cardiomyocytes (29, 105, 106) and similarly reduce cardiac Rac1 activity and oxidative stress in human heart failure (23). Indeed, the efficacy of statin drugs in heart failure treatment is thought to be mediated in part through repression of Rac1 *via* inhibition of prenylation and antagonism of maladaptive Rac1 signaling and oxidative stress (30, 105, 107, 108, 109). Inhibition of Rac1 S-palmitoylation may thus provide an alternative therapeutic approach for cardiac disease treatment by inhibiting maladaptive Rac1 signaling at the sarcolemma.

## Experimental procedures

### Animals

Cardiomyocyte-specific transgenic mice overexpressing zDHHC3 were generated by subcloning mouse *Zdhhc3* cDNA (Dharmacon, #MMM1013-202763213) into the re-engineered tetracycline-inducible αMHC promoter expression vector that permits tetracycline/doxycycline-extinguishable expression in the presence of a second transgene expressing the tTA expressed by the unmodified αMHC promoter expression vector (51). The DNA construct was digested with Not I restriction endonuclease and the promoter-cDNA fragment gel purified for oocyte injection at the Cincinnati Children’s Hospital Transgenic Animal and Genome Editing Core Facility as described previously (110, 111). Enzymatically dead *Zdhhc3* mutant transgenic mice overexpressing *Zdhhc3*^*DHHS*^ in cardiomyocytes were generated by site-directed mutagenesis of the α-MHC-*Zdhhc3* promoter-transgene construct using the QuikChange II XL Site- Directed Mutagenesis Kit (Agilent) to encode a mutation of Cys-157 in mouse zDHHC3 protein to Ser. Primers used for mutagenesis were Forward 5′-GCAAGATGGATCACCACAGTCCTTGGGTCAACAAC-3′ and Reverse 5′-GTTGTTGACCCAAGGACTGTGGTGATCCATCTTGC-3′. Transgenic mice were generated on the FVB/N genetic background. To induce transgene expression in the adult heart, transgenic mice were bred on doxycycline-containing chow (625 mg/kg diet, Cincinnati Lab Supply, #TD1811541) to repress transgene expression until 3 weeks of age when experimental mice were weaned from the dams and placed on a normal lab chow diet. All molecular analyses were performed in the high-expressing line of *Zdhhc3* transgenic mice 2 months following induction of transgene expression (removal of doxycycline) in the adult heart unless otherwise stated.

Cardiac-specific *Zdhhc3* gene–deleted mice were generated as previously described (112), using embryonic stem cells with a knockout first allele of the *Zdhhc3* gene (*Zdhhc3*^*tm1a(EUCOMM)Hmgu*^) obtained from the European Mouse Mutant Cell Repository that was used in aggregation with 8-cell embryos to generate chimeric mice. Germline male chimeras were then crossed with *Rosa26-FLPe* females (Jackson Laboratory, #9086) to remove the neomycin cassette and generate a conditional allele with loxP (f) sites flanking exon 3 of *Zdhhc3*. Cardiac-specific deletion of *Zdhhc3* was achieved by crossing *Zdhhc3-loxP* mice with mice containing the *Nkx2.5cre* allele (*B6129S1-Nkx2-5*^*tm1(cre)Rjs/J*^, JAX strain 030047). *Rac1*^*f/f*^ (JAX; strain 005550) (113) and *Lgals1* gene-targeted (JAX; strain 006337, *C57BL/6NJ* background) (114) mice were obtained from Jackson Laboratory. Rac1 cardiac-specific deletion mice were generated by crossing *Rac1*^*f/f*^ mice with Tg(Myh6-cre [called αMHCcre here])1Jmk mice (JAX stock #009074) (115), and because the αMHCcre transgene is on the X-chromosome only, male mice were used to avoid chimerism due to X-linked inactivation. *Gnaq-Gna11*– (72) (Gα_q_ and Gα_11_ proteins, respectively) targeted mice were also employed. *Zdhhc7*- (116) targeted mice were characterized and previously described. All mice were in the C57BL/6J background unless otherwise noted.

AAV9 was generated by subcloning full-length mouse *Zdhhc* cDNAs (kind gift of Dr Masaki Fukata, National Institute for Physiological Sciences, such as *Zdhhc3, 5, 6, 7, 13*) (41) with 2× hemagglutinin (HA) epitopes on the N terminus into the pAAV-MCS vector (Agilent) and AAV9 was produced by Vigene. Mouse pups were injected in the chest cavity at postnatal day 6 with 1 × 10^12^ viral genomes of the indicated AAV9 as described previously (117) with the exception of *Zdhhc3* and *Zdhhc7* that were injected at lower doses of 0.5 × 10^12^ or 1 × 10^11^ viral genomes per pup, respectively, due to lethal cardiomyopathy associated with robust expression. Controls were injected with 1 × 10^12^ viral genomes of empty AAV9 vector or sterile 1× PBS. AAV studies were performed in CD1 mice with the exception of Figure S1 studies that were performed in the FVB/N genetic background.

Echocardiography was performed as described previously (118, 119). Mortality was defined as a mouse being found dead in the cage or veterinarian-recommended euthanasia due to symptoms of congestive heart failure.

### Ethics approval and rigor

All animal procedures were approved by the Cincinnati Children’s Hospital Institutional Animal Care and Use Committee and conformed to the Guide for the Care and Use of Laboratory Animals of the National Institutes of Health in the USA. Randomization of mouse groups was not performed given that they were genetically identical and the same ages of mice were used in comparison studies. ARRIVE guidelines were followed in all mouse experimentation. No human materials or subjects were used. Blinding of animal groups was performed where possible. No data were excluded in the analysis of all figures and tables used in this report.

### Pathological hypertrophy models

Transverse aortic constriction procedures were performed as previously described (119). Briefly, two-month-old mice were anesthetized with 3% isoflurane and intubated with an 18-gauge catheter. During surgery, mice were continuously anesthetized using a mouse ventilator (SomnoSuite, TSE Systems) at 1.7% isoflurane. Mice were thoracotomized, followed by isolation of the transverse aorta. Constriction of the transverse aorta was achieved by tying a suture around both the transverse aorta and a 26-gauge needle; the needle was removed to generate the constriction. The thoracic incision was sutured and sealed with GLUture (Zoetis, Butler Schein, #034418). Post extubation, mice were treated with sustained-release buprenorphine (0.2 mg/kg) injected subcutaneously. For chronic angiotensin-II infusion, two-month-old mice were subcutaneously implanted with osmotic pumps containing saline or AngII (3 μg/g/day, Alzet #1002) for 2 weeks. Mice and incisions were monitored daily following surgery. Sham surgeries or installation of saline pumps were performed in the same manner. Hearts were harvested for the indicated times after surgery.

### Western blotting, immunoprecipitations, membrane fractionation, and GTPase activity

For evaluation of small GTPase activity and small GTPase protein levels, hearts were homogenized in assay buffer (25 mM Hepes pH 7.5, 150 mM NaCl, 1% NP-40, 10 mM MgCl_2_, 1 mM EDTA, and 2% glycerol) with protease inhibitors (Roche) and lysates were cleared by centrifugation. RhoA activity was evaluated by affinity purification of RhoA-GTP using rhotekin agarose beads (Cell Biolabs), and the activity of Rac1 and Cdc42 were assessed by affinity purification using magnetic beads coupled to the p21-binding domain of PAK (Millipore) that specifically binds the active (GTP-bound) forms of Rac1 and Cdc42. Following affinity purification, GTP-bound small GTPases were eluted from beads for SDS-PAGE by boiling in Laemmli buffer.

Western blotting was performed as described previously. Mouse hearts were homogenized in radioimmunoprecipitation assay (RIPA) buffer (50 mM Tris•HCl pH 7.4, 1% Triton X-100, 1% sodium deoxycholate, 1 mM EDTA, 0.1% SDS) containing Halt protease and phosphatase inhibitor cocktail (Thermo Fisher Scientific 78442) and then sonicated, clarified by centrifugation, and boiled in Laemmli buffer. For detection of zDHHC proteins, RIPA lysates were used without boiling (Fig. S2) or cardiac lysates were made in 50 mM Tris•HCl pH 7.6, 10 mM Na_4_PO_2_O_7_•10H_2_O, 6 M urea, 10% glycerol, and 2% SDS. Biochemical fractionation of mouse hearts into membrane and cytosolic fractions was performed exactly as described elsewhere (120). HA-tagged zDHHC proteins were immunoprecipitated from cardiac lysates with anti-HA magnetic beads (Pierce, #88836) and eluted by boiling in Laemmli buffer. Samples were separated by SDS- PAGE and transferred to polyvinylidene difluoride membranes (Millipore Immobilon-FL, #IPVH00010) for immunoblotting. Polyvinylidene difluoride membranes were blocked in 5% dry milk diluted in Tris-buffered saline with 0.1% tween-20 (TBST), incubated with primary antibodies diluted in 5% milk in TBST overnight at 4 °C followed by incubation with LiCor IRDye secondary antibodies diluted 1:10,000 in 5% milk in TBST with 0.02% SDS for 2 h at room temperature, and imaged on a Li-Cor Odyssey CLx imaging system. Primary antibodies used were calnexin (Abcam, #ab22595, 1:1000), Cdc42 (Abcam, #ab64533, 1:500), phos-ERK1/2 (Cell Signaling Technology, #4370, 1:500), ERK1/2 (Cell Signaling Technology, #9102, 1:500), galectin-1 (Abcam, #EPR3205, 1:1000), GAPDH (Fitzgerald, #10R-G109A, 1:50,000), HA (Abcam, #ab9110, 1:1000), integrin β1D (Millipore, #MAB1900, 1:1000), PAK1 (Cell Signaling Technology, #2602, 1:500), Rac1 (BD Transduction Laboratories, #610650, 1:500), pan-Ras (Thermo Fisher Scientific, #MA1-012, 1:1000), H-Ras (Santa Cruz Biotechnology, #sc-29, 1:500), RhoA (Cell Signaling Technology, #2117, 1:500), RhoGDIα (BD Transduction Laboratories, #610255, 1:4000), α-tubulin (Sigma, #T5168, 1:1000), zDHHC3 (Abcam, #ab31837, 1:500), and zDHHC7 (Abcam, #ab138210, 1:500).

### Immunocytochemistry

Immunocytochemistry was performed on adult cardiomyocytes in suspension exactly as described previously (111). Cardiomyocytes were isolated from mouse hearts by Langendorff perfusion, fixed with 4% paraformaldehyde for 15 min at room temperature, incubated in blocking solution (1× PBS, 5% goat serum, 1% bovine serum albumin, 1% glycine, 0.2% Triton X-100) for 1 h at room temperature, and then immunostained with Rac1 (BD Transduction Laboratories, #610650), zDHHC3 (Abcam, #ab31837), or galectin-1 (Abcam, #ab58085). Primary antibodies diluted 1:50 in blocking solution overnight at 4 °C. Cardiomyocytes were then washed in 1× PBS with 0.1% NP-40, incubated with Alexa Fluor secondary antibodies (Molecular Probes) diluted 1:1000 in blocking solution for 2 h at room temperature, washed again in 1× PBS with 0.1% NP-40, and mounted on slides with Prolong Diamond Antifade Mountant with 4′,6-diamidino-2-phenylindole (Molecular Probes). Imaging was performed using a Nikon A1 Confocal microscope.

### Acyl-RAC and mass spectrometry

S-palmitoylated proteins were purified from cardiac lysates by Acyl-RAC as described previously (54). Briefly, cardiac lysates were made in RIPA buffer as described above, diluted with 100 mM Hepes pH 7.4, 1 mM EDTA to a concentration of 2.5% SDS, and free thiols were blocked with 0.2% methyl methanethiosulfonate at 42 °C for 20 min. Proteins were then acetone precipitated at −20 °C and samples centrifuged for 10 min at 10,000*g*. Protein pellets were washed four times in 70% ice cold acetone to remove excess methyl methanethiosulfonate and protein pellets were dried and solubilized in 100 mM Hepes pH 7.4, 1% SDS, and 1 mM EDTA with protease inhibitors at 37 °C. Protein concentration was quantified and samples diluted to an equal concentration. For affinity purification of S-palmitoylated proteins, 450 μl lysate was combined with 200 μl of 100 mM Hepes pH 7.4 with 1 mM EDTA, 300 μl of 1M NH_2_OH pH 7.4 or 150 mM Tris•HCl pH 7.4 as a negative control, and 30 μl thiopropyl sepharose (Sigma) and incubated at room temperature for 3 h. Thiopropyl sephaarose beads were then washed four times in 100 mM Hepes pH 7.4, 0.3% SDS with 1 mM EDTA, and S-palmitoylated proteins were eluted from thiopropyl sepharose by boiling in Laemmli buffer.

Stable NIH3T3 cells (ATCC CRL-1658, certified *mycoplasma* free and authentic) overexpressing zDHHC3 or GFP as a control were generated using the pLVX lentiviral system (Clontech). Cells were labeled by SILAC by passaging at least 9 times in Dulbecco's Modified Eagle Medium for SILAC lacking lysine and arginine (Thermo Fisher Scientific) containing 10% dialyzed fetal bovine serum (Thermo Fisher Scientific) and supplemented with [^13^C_6_, ^15^N_2_] L-lysine and [^13^C_6_, ^15^N_4_] L-arginine (Thermo Fisher Scientific) for “heavy” 3T3-*Zdhhc3* cells or normal L-lysine and L-arginine (Thermo Fisher Scientific) for “light” 3T3-eGFP cells. Mass spectrometry sequencing of S-palmitoylated peptides was performed essentially as described previously (54) with slight modifications. Protein extracted from SILAC-labeled 3T3-*Zdhhc3* and 3T3-eGFP cells was mixed 1:1, and S-palmitoylated proteins were purified by Acyl-RAC as described. Following the final wash, thiopropyl sepharose beads were incubated overnight at 37 °C with 2 μg trypsin Gold (Promega, #V5280) in 50 mM NH_4_HCO_3_, 1 mM EDTA. After on-resin trypsin digestion, thiopropyl sepharose beads were washed five times in 100 mM Hepes, 1% SDS, 1 mM EDTA, then washed four times in 10 mM NH_4_HCO_3_, and once in 50 mM NH_4_HCO_3_. Captured peptides were then eluted from thiopropyl sepharose by incubation with 100 mM DTT (Roche) in 50 mM NH_4_HCO_3_ at 70 °C for 45 min and further processed for mass spectrometry sequencing at the University of Cincinnati Proteomics Laboratory (121). Eluted peptides were alkylated with 200 l of 400 mM iodoacetamide in 25 mM NH_4_HCO_3_ at 37 °C for 2 h and loaded onto C18 stage tips made from 3M Empore extraction disks, washed twice with 50 μl of 0.1% formic acid, and stage tips were then eluted three times with 50 μl of 80% acetonitrile/0.1% formic acid by centrifuging through the column at 1600*g* for 5 min. Elutions were pooled, dried, and reconstituted in 6 ml of 0.1% formic acid, and peptides were sequenced by nanoscale liquid chromatography coupled to tandem mass spectrometry and searched using the Protein Pilot program (Sciex).

Mouse embryonic fibroblasts were isolated as described previously (122) from homozygous *Zdhhc3* loxP-targeted littermate embryos at approximately embryonic day 12, immortalized by lentiviral transduction with large T antigen (123), and then transduced with recombinant adenovirus to express beta-galactosidase as a control or Cre recombinase to delete *Zdhhc3*. SILAC labeling, Acyl-RAC, and mass spectrometry sequencing were then performed as described above.

### Acyl biotin exchange

Acyl biotin exchange was performed as previously described (94, 124). Briefly, hearts were minced in 1% β-D-maltoside in 1× PBS, supplemented with HALT protease-phosphatase inhibitors and ML211 (acyl protein thioesterases inhibitor, 10 mM, Cayman Chemicals), and mechanically homogenized (Omni Tissue Master 125). Lysates were spun down at 21,000*g* for 30 min at 4 °C. Equal quantities of proteins were incubated with 50 mM N-ethylmaleimide (Thermo Fisher Scientific, 23030) overnight at 4 °C on a rotator. Samples underwent three rounds of precipitation with chloroform-methanol, followed by incubation with freshly made 400 mM hydroxylamine pH 7 (Sigma 159417) and 1 mM biotin-HPDP (Cayman Chemicals 16459) for 50 min at 37 °C with gentle mixing. Negative controls were incubated with 400 mM NaCl. Samples underwent an additional three rounds of chloroform-methanol precipitation followed by incubation with streptavidin agarose with gentle mixing overnight at 4 °C. Beads were then washed four times with wash buffer (150 mM NaCl, 50 mM Tris pH7, 5 mM EDTA, 0.2% TritonX-100, 0.1% SDS) and eluted with elution buffer (400 mM Tris pH 6.8, 40% glycerol, 1% bromophenol blue, 5% SDS) for 15 min at 80 °C with mixing. Eluted proteins are run on SDS gels followed by transfer to nitrocellulose (Bio-Rad 162-0112) and Western blotting. Palmitoylation levels of calnexin were used as a control (94).

### Statistical analyses

All statistical analyses were performed using GraphPad Prism (graphpad.com) with a *p*-value < 0.05 considered significant. Tests between two groups with only one variable were conducted with unpaired t-tests. Analysis between more than two groups were analyzed by a one- or two-way ANOVA with Holm Sidak’s multiple comparison test for post hoc pairwise comparisons. Data are reported at mean ± SEM.

## Data availability

The datasets generated during and/or analyzed during the current study are available in this study or the supporting information that are provided.

## Supporting information

This article contains supporting information.

## Conflict of interest

The authors declare that there are no conflicts of interests with the contents of this article.
