# Supplementary material for: Palmitoylation-dependent regulation of cardiomyocyte Rac1 signaling activity and minor effects on cardiac hypertrophy
Source: J Biol Chem. 2023 Nov 3;299(12):105426. doi: 10.1016/j.jbc.2023.105426 (PMC10716590; doi:10.1016/j.jbc.2023.105426)
Supplement: Supplemental Table S1 [file mmc1.pdf]

Supporting Table 1. zDHHC3 protein substrates with possible cardiac function

3T3-eGFP (light) vs 3T3 *Zdhhc3* (heavy)		
**Protein ID**	**Peptide**	**H:L**
Desmoplakin (Dsp)	LLEAQAC^{2668} TGGIIHPTTGQK	5.46
Poly (rC)-binding protein 1 (Pcbp1)	LVVPATQC^{109}GSLIGK	5.44
ATP-dependent RNA helicase (Ddx5)	LIDFLEC^{234}GK	2.99
Myb-binding protein 1A (Mybbp1a)	SPAESC^{614}DVLGDIQTC^{623}IKK	2.91
Protein scribble homolog (Scrib)	HC^{22}SLQVVPEEIYR	2.02
Protein deglycase DJ1 (Park7)	VTVAGLAGKDPVQC^{46}SR	1.73
Galectin-1 (Lgals)	FNAHGDANTIVC^{61}NTK	1.72
Galectin-1 (Lgals)	DSNNLC^{43}LHFNPR	1.80
Galectin-1 (Lgals)	AC^{3}GLVASNLNLKPGEC^{17}LK	1.66
Filamin A	IVSPSGAAVPC^{1018}KVEPGLGADNSVVR	1.58
Delta-catenin	YQEALPTVANSTGPHAASC^{618}FGAK	1.49
Rac1	AVLC^{178}PPPVK	1.32
Phosphatidylinositol 4-kinase 2 alpha	LC^{174}C^{175}PC^{177}C^{178} FGR	1.23
**WT MEFs (light) vs *Zdhhc3* KO MEFs (heavy)**		
**Protein ID**	**Peptide**	**H:L**
Galectin-1 (Lgals)	AC^{3}GLVASNLNLKPGEC^{17}LK	0.42
Gα (q) subunit	TLESIMAC^{9}C^{10}LSEEAKEAR	0.43
Tetraspanin-14	EKCGVPFSC^{186}C^{187}VPDPAQK	0.57
Gα (11) subunit	MTLESMMAC^{9}C^{10}LSDEVKESK	0.59
Caveolin-2	SVTDVVIGPLC^{145}TSVGR	0.61

Table S1. **Identification of Rac1 as a novel substrate of zDHHC3**.
The table shows selected S-acylated peptides and proteins enriched in recombinant NIH3T3-*Zdhhc3* cells compared to NIH3T3-eGFP controls or reduced in *Zdhhc3* gene-deleted mouse embryonic fibroblasts (MEFs) with known functions in cardiac hypertrophy or established zDHHC3 substrates. Full data set is in Supporting data files 1 and 2. Putative zDHHC3-modified peptides with heavy:light ratio (H:L) >1.0 are shown above given the overexpression strategy of *Zdhhc3* and those <1.0 (reduced S-acylation) are shown below in the absence of *Zdhhc3* (KO cells). Data are only shown for peptides sequenced multiple times.
