# Supplementary material for: Palmitoylation-dependent regulation of cardiomyocyte Rac1 signaling activity and minor effects on cardiac hypertrophy
Source: J Biol Chem. 2023 Nov 3;299(12):105426. doi: 10.1016/j.jbc.2023.105426 (PMC10716590; doi:10.1016/j.jbc.2023.105426)
Supplement: Supplemental Figs. S1–S8 [file mmc2.pdf]

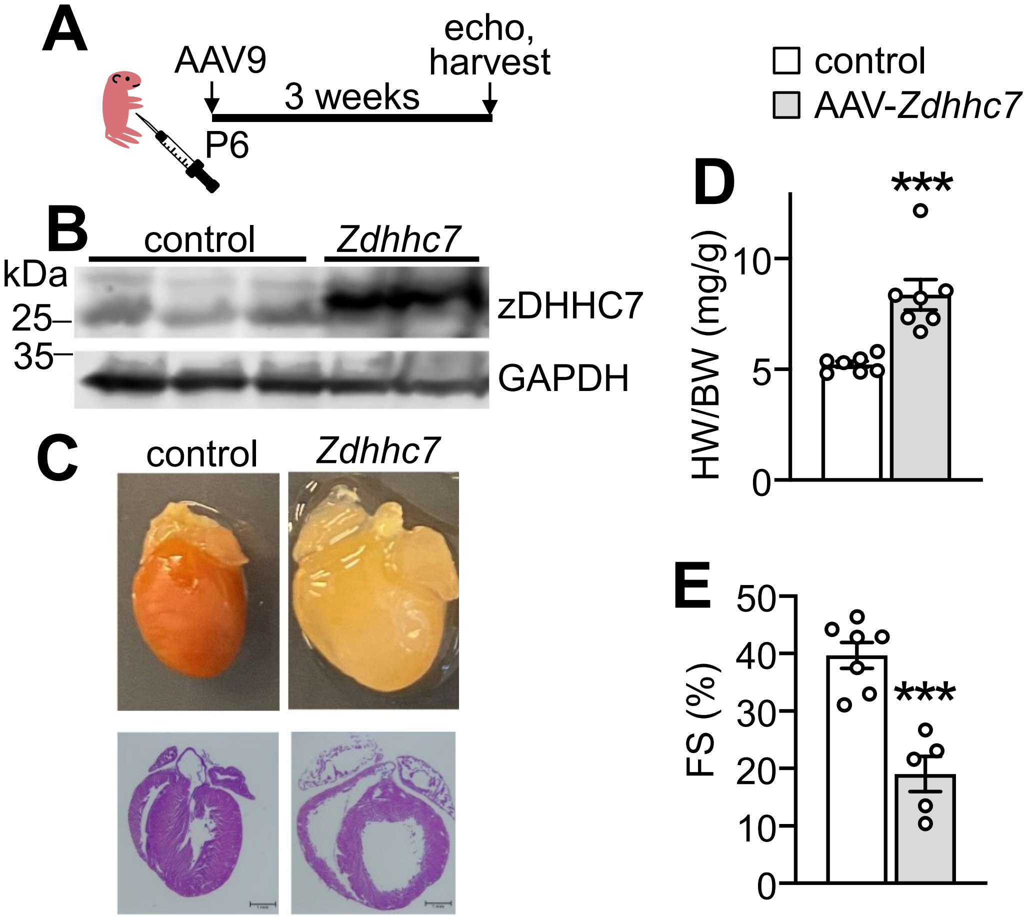

Figure S1. **Overexpression of *Zdhhc7*, the most closely related S-acyl transferase to *Zdhhc3*, also causes cardiomyopathy**. *A,* Experimental schematic and *B,* western blotting for AAV9-mediated overexpression of *Zdhhc7* in the hearts of mice injected at day 6 after birth (P6) and harvested 3 weeks later for analysis. GAPDH is used as a tissue processing and protein loading control. *C,* Whole hearts and H&E-stained cardiac histological sections after 3 weeks of AAV9-mediated expression of the *Zdhhc7* cDNA, scale bar = 1 mm for the H&E images that correspond to the same hearts above for sizing reference. *D,* Heart weight-to-body weight ratios, n=7, unpaired t-test (p=0.0007) *E,* and fractional shortening (FS) as assessed by echocardiography in mice with cardiac overexpression of *Zdhhc7*, 3 weeks after recombinant AAV9 injection. n=5-7, unpaired t-test (p=0.0002). Error bars throughout the figure panels represent mean ± SEM.

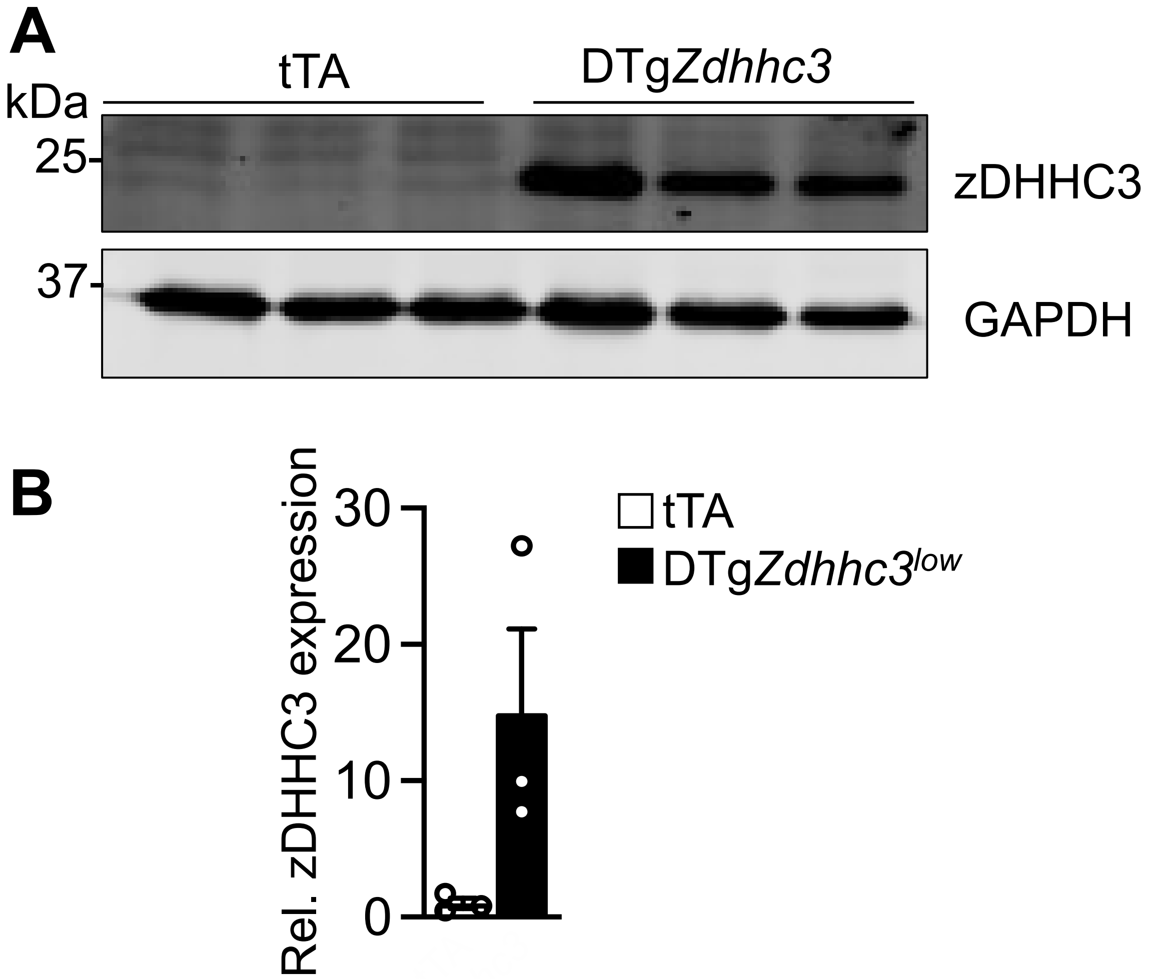

Figure S2. Transgenic overexpression of zDHHC3.
A, Western blotting and *B,* quantification of zDHHC3 protein levels in hearts of the low expressing line of cardiomyocyte-specific *Zdhhc3* transgenic mice at 2 months of age. Corresponding phenotypic data are in Fig 2*D-I*. GAPDH was run as a loading and protein processing control.

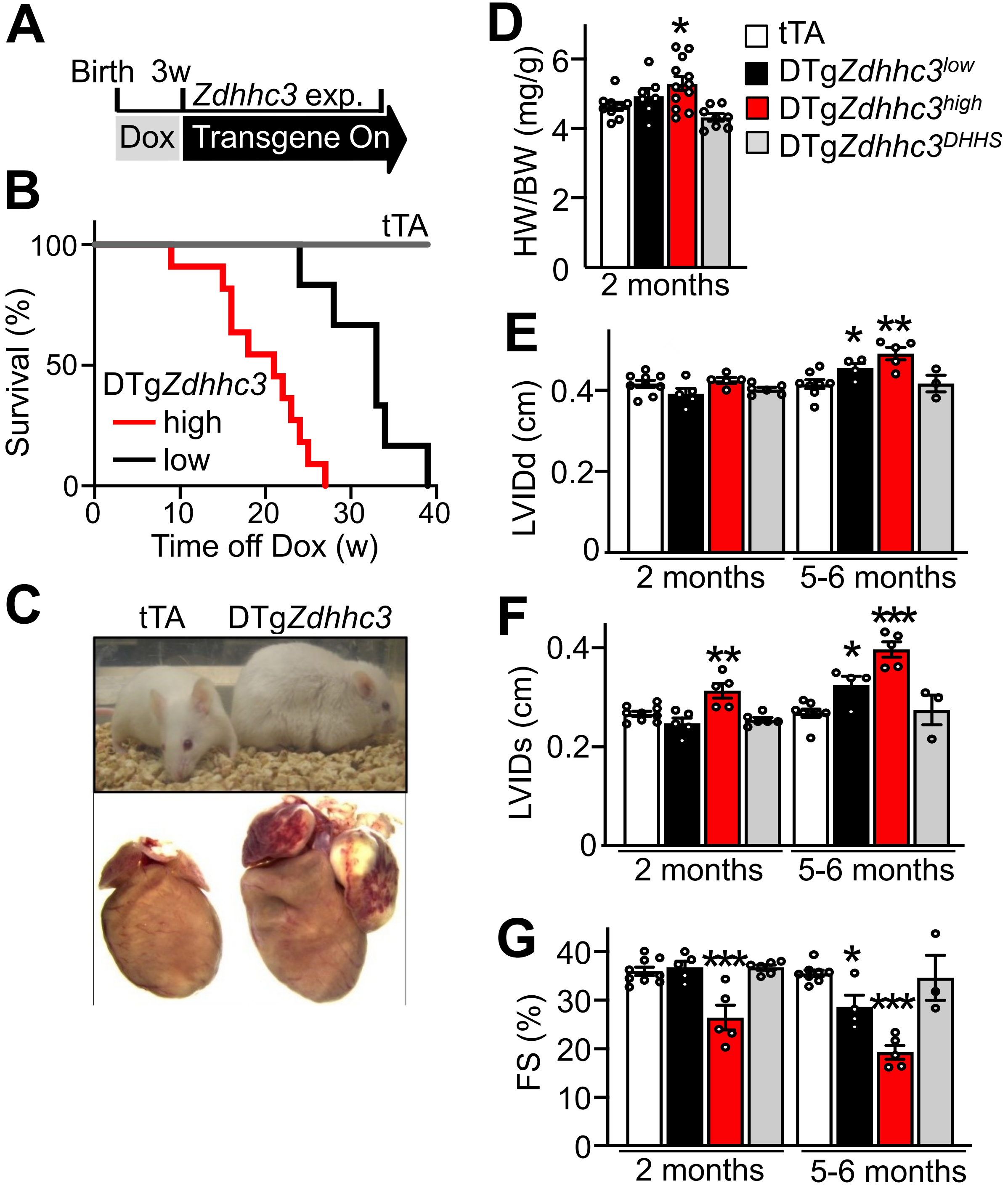

Figure S3. Overexpression of *Zdhhc3* in the adult heart results in congestive heart failure. *A,* Schematic of transgene expression strategy where mice were kept on Dox food until weaning and then switched to lab chow to induce expression. *B,* Kaplan-Meier survival curve. n= 9 tTA, 6 DTgZ*dhhc3*^{low}, 11 DTg*Zdhhc3*^{high}. Survival curve log-rank test (p<0.0001). *C,* Pictures of adult DTg*Zdhhc3* and littermate tTA control mice in a cage (low line), along with whole hearts imaged together for comparison with no scale bar needed. *D,* Heart weight-to-body-weight ratios after 2 months of transgene expression in the indicated groups of mice. n=7-12. 1-way ANOVA (p=0.002) with pairwise comparison of tTA and DTg*Zdhhc3*^{high} (p=0.02). *E-G,* Echocardiographic measurement of *E,* diastolic left ventricular inner diameter in diastole (LVIDd), 2-way ANOVA showed a main effect of genotype (p=0.001) and an interaction (p=0.007). *F,* systolic LVID (LVIDs), 2-way ANOVA showed a main effect of genotype (p<0.0001) and an interaction (p=0.0009). *G,* fractional shortening (FS%) at the indicated time after transgene induction in the indicated lines of mice. 2-way ANOVA showed a main effect of genotype (p<0.0001) and an interaction (p=0.035). High line Tg*Zdhhc3* mice were assayed by echocardiography at 5 months after transgene expression due to excessive mortality by 6 months as shown in *B.* n=3-8. Error bars throughout the figure panels represent mean ± SEM. *P<0.05, **P<0.005, ***P<0.0001

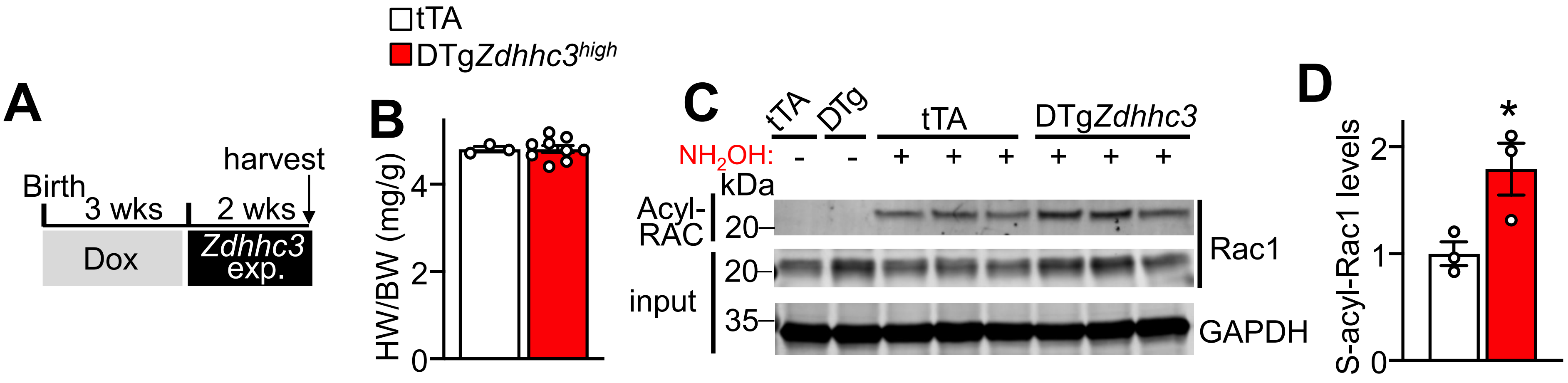

Figure S4. **Induction of Rac1 palmitoylation precedes cardiac hypertrophy and failure in DTgZ*dhhc3* mice (high line).** *A,* Experimental design with transgene induction at 3 weeks of age when Dox was removed from the diet and mice were harvested 2 weeks later for analyses of protein palmitoylation. *B,* Heart weight-to-body weight ratios. n=3-9 in the 2 indicated groups of mice. Unpaired t-test (p=0.99) *C,* Western blotting for palmitoylated and total Rac1 in transgenic hearts at harvest for the indicated groups of mice. *D,* Quantification of palmitoylated Rac1 normalized to GAPDH expression from *C*. n=3. Unpaired t-test (p=0.04) Error bars throughout the figure panels represent mean ± SEM. *P<0.05.

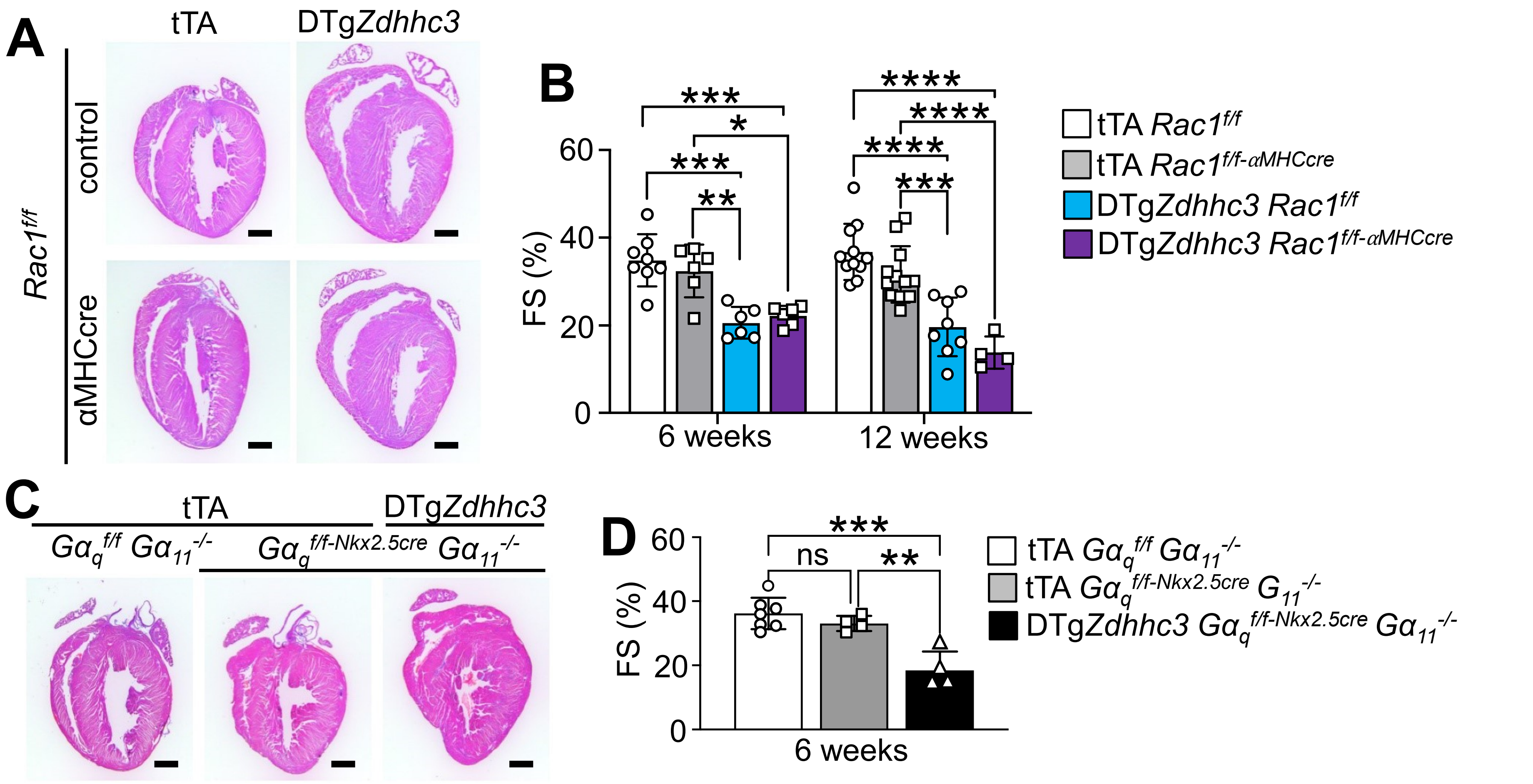

Figure S5. **Deletion of Rac1 or double deletion of the genes encoding Gαq/α11 does not rescue DTg*Zdhhc3* dependent cardiac maladaptation**. *A*, Representative H&E images of cardiac histological sections from single transgenic controls (tTA *Rac1*$^{f/f}$ and tTA *Rac1*$^{f/f\text{-}\alpha MHCcre}$) or low line double transgenic (DTg*Zdhhc3* Rac1$^{f/f}$ and DTg*Zdhhc3 Rac1*$^{f/f\text{-}\alpha MHCcre}$) at 12 weeks, scale bar = 500 µm. *B,* Echocardiography measured fractional shortening (FS%) at the indicated time after transgene expression. n=4-9. 2-way ANOVA showed a main effect of genotype ($p<0.0001$) but no interaction ($p=0.17$). *C,* Representative H&E-stained cardiac histological images from single transgenic controls (*tTA* $G\alpha_q^{f/f} G\alpha_{11}^{-/-}$ and tTA $G\alpha_q^{f/f\text{-}Nkx2.5cre}\ G\alpha_{11}^{-/-}$) or low line double transgenic (DTg*Zdhhc3* $G\alpha_q^{f/f\text{-}Nkx2.5cre}\ G\alpha_{11}^{-/-}$). Scale bar = 500 µm. *D,* echocardiography measured fractional shortening (FS%) at 8 weeks. n=4-7. 1-way ANOVA ($p=0.0002$) with pairwise comparisons test of *tTA* $G\alpha_q^{f/f} G\alpha_{11}^{-/-}$ versus tTA $G\alpha_q^{f/f\text{-}Nkx2.5cre}\ G\alpha_{11}^{-/-}$ ($p=0.30$), *tTA* $G\alpha_q^{f/f} G\alpha_{11}^{-/-}$ versus DTg*Zdhhc3* $G\alpha_q^{f/f\text{-}Nkx2.5cre}\ G\alpha_{11}^{-/-}$ ($p=0.0002$), and tTA $G\alpha_q^{f/f\text{-}Nkx2.5cre}\ G\alpha_{11}^{-/-}$ versus DTg*Zdhhc3* $G\alpha_q^{f/f\text{-}Nkx2.5cre}\ G\alpha_{11}^{-/-}$ ($p=0.002$) . Z3 (*Zdhhc3*). DTg*Zdhhc3* alone controls are shown previously in Figure 2*I*. Error bars throughout the figure panels represent mean ± SEM. *P<0.05, **P<0.01, ***P<0.001, ****P<0.0001.

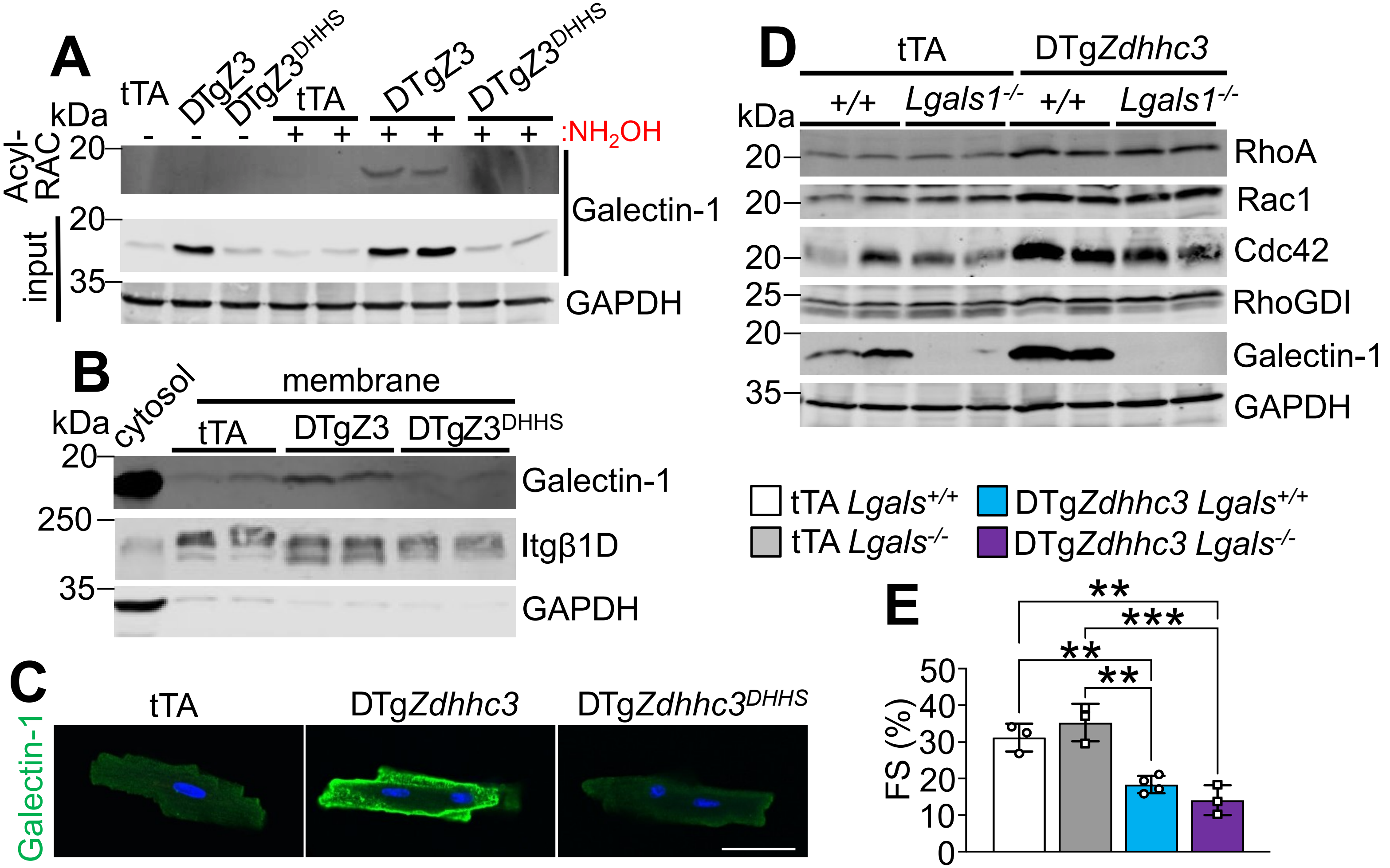

Figure S6. **Galectin-1 palmitoylation in the heart by zDHHC3**. *A,* Western blotting for palmitoylated galectin-1 purified by Acyl-RAC. (-) indicates negative controls lacking NH_2OH treatment. Input of galectin-1 is shown as well as GAPDH as a tissue processing and loading control. *B,* Western blotting for the indicated membrane proteins isolated from the indicated transgenic mouse hearts at 2 months of age. Itgβ1D, integrin β1D, shows membrane preparation purity. GAPDH is a tissue processing and loading control. *C,* Immunocytochemistry for endogenous galectin-1 (green) in adult cardiomyocytes isolated from the indicated transgenic hearts at 2-months of age (6 weeks of transgene expression). Scale bar = 50 µm. *D,* Western blotting for small GTPases and galectin-1 from hearts of the indicated mice at 2 months of age (6 weeks of transgene expression). GAPDH controls for tissue processing and blot loading. *E,* Echocardiography measured fractional shortening (FS%) of hearts from the indicated mice at 3 months, transgene expression began at weaning. n=3-4., 2-way ANOVA showed a main effect of DTgZ3 overexpression ($p<0.0001$) but no interaction ($p=0.08$). Error bars represent mean ± SEM. Z3, *Zdhhc3* targeted. **P<0.01, ***P<0.001

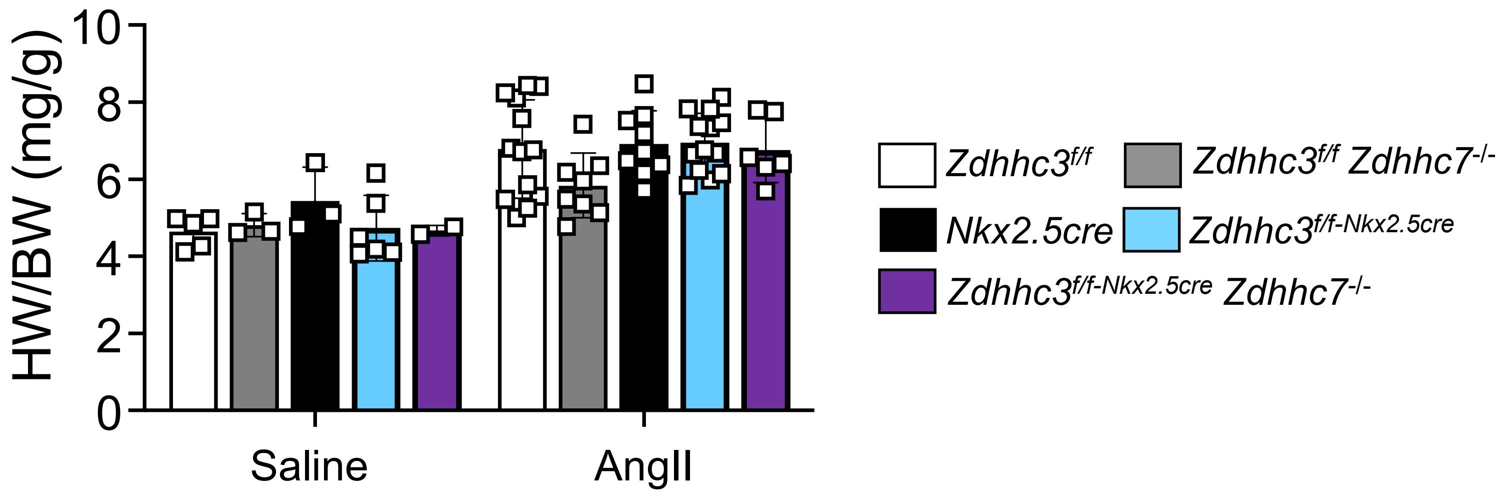

Figure S7. *Zdhhc3* and *Zdhhc7* are not required for cardiac hypetrophy in response to chronic angiotensin-II (AngII) treatment. Heart weight-to-body weight ratios of *Zdhhc3*$^{f/f}$, *Zdhhc7*$^{-/-}$ *Zdhhc3*$^{f/f}$, *Nkx2.5cre*, *Zdhhc3*$^{f/f\text{-}Nkx2.5cre}$, and *Zdhhc7*$^{-/-}$ *Zdhhc3*$^{f/f\text{-}Nkx2.5cre}$ mice after 2 weeks of AngII infusion (3 mg/kg/day). Saline: n=2-6, AngII: n= 6-13. Two-way ANOVA revealed a main effect of AngII treatment ($p<0.0001$) but no interaction ($p=0.51$). Error bars represent mean ± SEM.

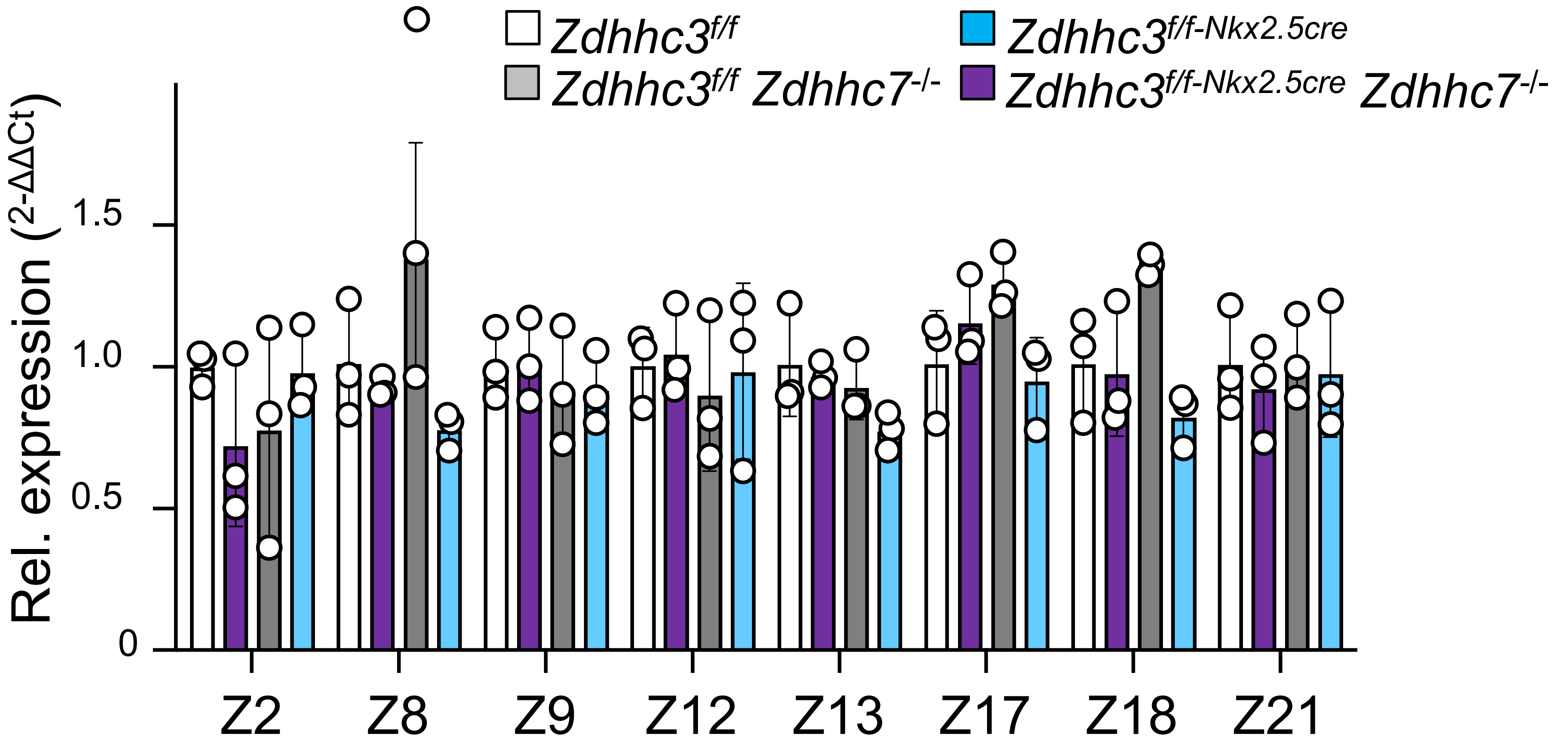

Figure S8. **Other *Zdhhc* genes do not compensate for loss of *Zdhhc3/7* in the heart after 2 months of TAC**. Relative expression of other Golgi-localized *Zdhhc* genes after 2 months of TAC, n=3. 2-way ANOVA showed no interaction (p=0.08). Z, *Zdhhc*. Error bars represent mean ± SEM.
